# Midwife-Led Versus Obstetrician-Led Perinatal Care for Low-Risk Pregnancy: A Systematic Review and Meta-Analysis of 1.4 Million Pregnancies

**DOI:** 10.3390/jcm13226629

**Published:** 2024-11-05

**Authors:** Shyamkumar Sriram, Fahad M. Almutairi, Muayad Albadrani

**Affiliations:** 1 Department of Rehabilitation and Health Services, College of Health and Public Service, University of North Texas, Denton, TX 76203, USA; 2 Health Holding Company, Ministry of Health, Jeddah 22234, Saudi Arabia; 3 Department of Family and Community Medicine and Medical Education, College of Medicine, Taibah University, Madinah 42353, Saudi Arabia

**Keywords:** midwife, obstetrician, childbirth, low-risk, meta-analysis

## Abstract

**Background:** The optimum model of perinatal care for low-risk pregnancies has been a topic of debate. Obstetrician-led care tends to perform unnecessary interventions, whereas the quality of midwife-led care has been subject to debate. This review aimed to assess whether midwife-led care reduces childbirth intervention and whether this comes at the expense of maternal and neonatal wellbeing. **Methods:** PubMed, Scopus, Cochrane Library, and Web of Science were systematically searched for relevant studies. Studies were checked for eligibility by screening the titles, abstracts, and full texts. We performed meta-analyses using the inverse variance method using RevMan software version 5.3. We pooled data using the risk ratio and mean difference with the 95% confidence interval. **Results:** This review included 44 studies with 1,397,320 women enrolled. Midwife-led care carried a lower risk of unplanned cesarean and instrumental vaginal deliveries, augmentation of labor, epidural/spinal analgesia, episiotomy, and active management of labor third stage. Women who received midwife-led care had shorter hospital stays and lower risks of infection, manual removal of the placenta, blood transfusion, and intensive care unit (ICU) admission. Furthermore, neonates delivered under midwife-led care had lower risks of acidosis, asphyxia, transfer to specialist care, and ICU admission. Postpartum hemorrhage, perineal tears, APGAR score < 7, and other outcomes were comparable between the two models of management. **Conclusions:** Midwife-led care reduced childbirth interventions with favorable maternal and neonatal outcomes in most cases. We recommend assigning low-risk pregnancies to midwife-led perinatal care in health systems with infrastructure allowing for smooth transfer when complications arise. Further research is needed to reflect the situation in low-resource countries.

## 1. Introduction

Several models of management are available for women during childbirth. Obstetricians are the primary providers of care for the majority of women during childbirth in North America. Other countries, like New Zealand, Australia, the United Kingdom, Ireland, and the Netherlands, apply additional models of care, including midwife-led care. There has been a constant debate about the optimal model of ante-, intra-, and postpartum care for women [1,2].

The obstetrician-led model of care is hospital-based with access to advanced medical equipment and readiness for various obstetric complications during childbirth. This model suits all pregnancy and childbirth risk levels, with an opportunity for advanced interventions. Although midwives often provide the actual management during childbirth, obstetricians are responsible for the management provided in the obstetrician-led model. This model could be called the biomedical model, as it focuses on the pathology and tries to reverse it [3]. Obstetricians usually apply technology and obstetric skills to minimize the risk of bleeding, infection, and fetal/neonatal adverse outcomes. However, some of the used technologies—such as continuous electronic fetal monitoring—might lead to unnecessary interventions [4]. Women desiring instant access to interventions such as pain management and cesarean section prefer this model of support [5,6]. In high-income countries particularly, this tendency for increased obstetric interventions is an issue of great concern [7,8].

Midwife-led care could be provided at home or in midwifery units (birth centers). Midwifery units are primarily run by midwives who provide perinatal care and transfer complicated cases as appropriate [9,10]. These are either alongside midwifery units (AMUs) located within the hospital or freestanding midwifery units (FMUs) located outside hospitals [11]. Nevertheless, this model of management is only suitable for low-risk pregnancies. Higher-risk pregnancies require a higher level of care, and the presence of an obstetrician and the hospital setting are a must [11,12,13]. For low-risk pregnancies as well, complications that necessitate transfer to obstetrician-led care might arise. In such cases, a smooth transfer system is needed in terms of effective communication along with a vehicle for transportation (for women delivering at home or FMUs). The midwife-led model of care affirms the idea of intervention-less natural childbirth [14]. Both women and midwives tend to believe that spontaneous labor onset, continuous support, no pain relief or labor augmentation, skin-to-skin contact, and exclusive breastfeeding on demand come with the best perinatal outcomes [15]. This tendency for de-medicalization of childbirth, however, should not be at the expense of maternal and fetal/neonatal wellbeing.

For the purpose of this review, low-risk pregnancy is generally defined as an uncomplicated singleton pregnancy in a cephalic-vertex presentation in a healthy woman. This definition, while broadly consistent, can vary slightly across different studies, encompassing factors such as maternal age, absence of pre-existing medical conditions, and a history of uncomplicated pregnancies. Understanding these variations is crucial as they influence the scope and applicability of the findings. It is worth mentioning that the distinction between obstetricians’ and midwives’ care models’ philosophy in regard to the medicalization of care is not exclusive. Many obstetricians advocate for the natural birth process, and many midwives might tend to over-intervene. Several studies were conducted to compare midwife-led and obstetrician-led models of care during childbirth in low-risk pregnancies. However, the inconsistency in studies’ designs and findings makes it difficult to reach a conclusion. Therefore, we conducted this systematic review and meta-analysis to summarize the evidence on whether midwife-led and obstetrician-led models of perinatal care for low-risk pregnancies vary in the intervention rate. Also, we aimed to assess whether there is a difference in maternal and neonatal outcomes between these two models of care.

## 2. Methods

We conducted this systematic review and meta-analysis in accordance with the Cochrane Handbook for Interventional Reviews [16]. After that, we reported our study following the Preferred Reporting Items for Systematic reviews and Meta-Analysis guidelines [17].

### 2.1. Information Sources

We conducted an electronic search in PubMed, Cochrane Library, Scopus, and Web of Science using the following strategy: (Midwife OR midwives OR “traditional birth attendant” OR “traditional birth attendants” OR Midwifery OR “Midwifery-Led” OR “midwife-led” OR “Home birth”) AND (physician OR doctor OR clinician OR “general practitioner” OR “medical officer” OR obstetrician OR specialist OR consultant OR “Obstetrician-Led” OR “obstetrician-gynaecologist-led” OR “physician-led”) AND (Birth OR childbirth OR parturition OR delivery OR deliveries OR labor OR pregnancies OR Pregnancy OR pregnant* OR Labour OR Childbearing) AND (“low risk” OR “low-risk”).

Databases were searched from 2000 until 26 June 2024, and no other filters were applied. After that, references of the included studies were manually screened for additional eligible studies.

### 2.2. Eligibility Criteria and Studies Selection

We included studies that compared midwife-led and obstetrician-led models of perinatal care during childbirth. Only studies enrolling healthy women with low-risk pregnancies were included. We excluded studies not published in English, conference abstracts, theses, books, and unpublished work.

Studies retrieved from database searches were screened after excluding duplicates. We started with title and abstract screening, followed by full-text screening for final eligibility. The screening was double-checked, and a discussion was conducted to resolve any inconsistent judgment.

### 2.3. Quality Assessment

The included Randomized Clinical Trials (RCTs) quality was evaluated using the Cochrane tool for risk of bias assessment. The tool assesses the quality of RCTs in terms of randomization and allocation process, blinding of participants, staff, and outcomes’ detectors, loss of participants, reporting of the pre-specified outcomes, and other possible sources of bias [18]. The National Institutes of Health tools were used to assess the risk of bias for cohort, cross-sectional, and case-control studies. The studies’ quality was rated as “good”, “fair”, or “poor” according to the score calculated from several questions on the studies’ methodology [19].

### 2.4. Data Extraction

We extracted data about the studies’ design, country, sample size, women’s eligibility criteria, and the place of birth for each group (midwife-led and obstetrician-led care). Baseline data about participants were also extracted from the studies. In particular, we extracted data about participants’ age, parity, body mass index (BMI), marital status, education, occupation, habits, gestational age, neonate sex, and neonatal birth weight.

The primary outcomes in this meta-analysis are incidences of unplanned cesarean section (CS), instrumental vaginal delivery (including forceps and venous delivery), epidural or spinal analgesia, postpartum hemorrhage (PPH) (defined as blood loss >500 mL for vaginal delivery and >1 L for CS), APGAR score < 7 (at one minute and five minutes), and intrapartum or neonatal mortality. Our secondary outcomes included incidences of birth interventions (CS because of suspected fetal distress, CS of non-progressive labor, augmentation of labor, NO2 or general anesthesia, local analgesia through pudendal nerve block, narcotics use, acupuncture pain relief, hydrotherapy pain relief, no pain relief, episiotomy, and physiological management of the third stage of labor), maternal outcomes (intact perineum, first or second-degree perineal tear, third or fourth-degree perineal tear, vaginal or labial tear, manual removal of the placenta, blood transfusion, maternal infection or fever, severe maternal morbidity, maternal intensive care unit (ICU) admission, the duration of labor (in hours), and the duration of hospital stay (in days)), and neonatal outcomes (one-minute mean APGAR score, five-minute mean APGAR score, umbilical cord arterial pH < 7.1, mean umbilical cord arterial pH, meconium-stained amniotic fluid, asphyxia, need for resuscitation, need for ventilation, shoulder dystocia, transfer to specialist neonatal care, neonatal ICU (NICU) admission, and breastfeeding initiation).

### 2.5. Quantitative Synthesis Methods and Assessment of Publication Bias

We conducted our meta-analyses using Review Manager (RevMan) software version 5.3 in the inverse variance method. We pooled continuous data using the mean difference (MD) and the 95% confidence interval (CI). For categorical outcomes, we used the risk ratio (RR) and the 95% CI in pooling data. Statistical significance was set at a *p*-value < 0.05. A chi-square *p*-value of <0.1 and an I^2^ value of ≥50% indicated significant heterogeneity in the results. For heterogeneous results, we conducted the meta-analysis via the random effect model instead of the default fixed effect model [20,21]. Moreover, we assessed the presence of publication bias using a funnel plot. The funnel plot is a scatter plot of the effect estimates from individual studies against their standard errors. In the absence of publication bias, the plot should resemble a symmetrical inverted funnel. However, asymmetry in the funnel plot can indicate potential publication bias. If the funnel plot revealed asymmetry, suggesting that smaller studies with negative or non-significant results may be underrepresented. This asymmetry highlights the need for caution when interpreting the results, as the observed effects might be influenced by the selective publication of studies with positive findings. It is important to note that any outcome with fewer than 10 studies could not be assessed for publication bias due to insufficient data.

## 3. Results

### 3.1. Studies Selection

**Figure 1:**
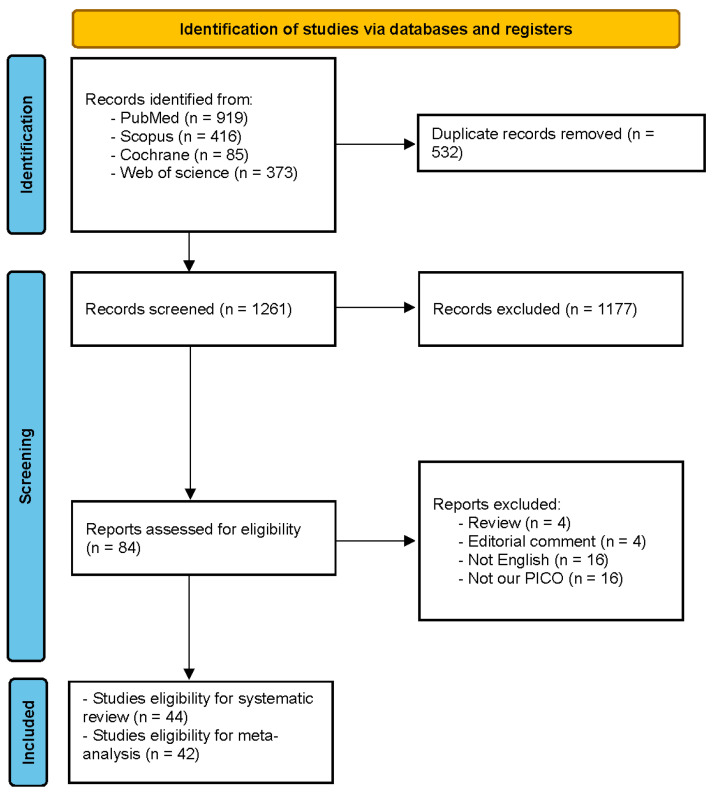
PRISMA flow diagram.

Our electronic database search retrieved 1793 results. Following duplicate exclusion, 1261 results remained for the title and abstract screening. Eighty-four full texts were reviewed for final eligibility, and a total of 48 articles reporting 44 studies were involved in this systematic review [22,23,24,25,26,27,28,29,30,31,32,33,34,35,36,37,38,39,40,41,42,43,44,45,46,47,48,49,50,51,52,53,54,55,56,57,58,59,60,61,62,63,64,65,66,67,68,69]. Two studies (Martin-Arribas et al. 2022 and Schroeder et al. 2017) were not included in the quantitative synthesis [25,42] (Figure 1).

### 3.2. Description of the Eligible Studies and Enrolled Women

We included three clinical trials [33,55,56,57,58], 36 cohort studies [22,23,24,27,28,29,30,31,32,34,35,36,37,38,39,40,41,42,43,44,45,46,47,48,49,52,53,59,60,61,62,63,64,65,66,67,68,69], three case-control studies [26,50,54], and two cross-sectional studies [25,51] in this review. These studies were conducted in different countries: Canada, the United States, Ireland, the United Kingdom, France, Germany, Spain, Norway, the Netherlands, Sweden, Belgium, Austria, Denmark, Nepal, Slovenia, Lithuania, China, Japan, Singapore, New Zealand, and Australia. The total number of women enrolled in this review is 1,397,320 (682,908 women received midwife-led care at home, FMU, or AMU, whereas 714,412 received obstetrician-led hospital-based care). Further description of the included studies’ methodology and main outcomes related to this review is available in Table 1.

Women enrolled in this review were around 30 years old and were mostly married or partnered. The majority of women had spontaneous onset of labor between 39 and 40 weeks of gestation and gave birth to a newborn weighing 3 to 3.6 kg. Table 2 provides a comprehensive description of the enrolled women’s baseline characteristics.

### 3.3. Quality Evaluation

The included clinical trials had a low risk of bias in all assessed aspects, with few exceptions. Pérez-Martínez et al. 2019 [33] applied non-random allocation of the participants, which adds a risk of selection bias. In addition, this study did not have a published protocol (Figures S1 and S2). All the included cohort and cross-sectional studies had good quality, indicating minimal risk of bias (Table S1). The included case-control studies were judged to provide fair evidence. Potential sources of bias in these studies were the non-random, non-concurrent selection of the controls and the lack of adjustment for the confounders (Table S2). The inability to blind the enrolled women, study personnel, and outcomes’ detectors might have added a potential source of bias in all the included studies.

### 3.4. Quantitative Synthesis Results

#### 3.4.1. Primary Outcomes

##### Unplanned Cesarean Section

**Figure 2:**
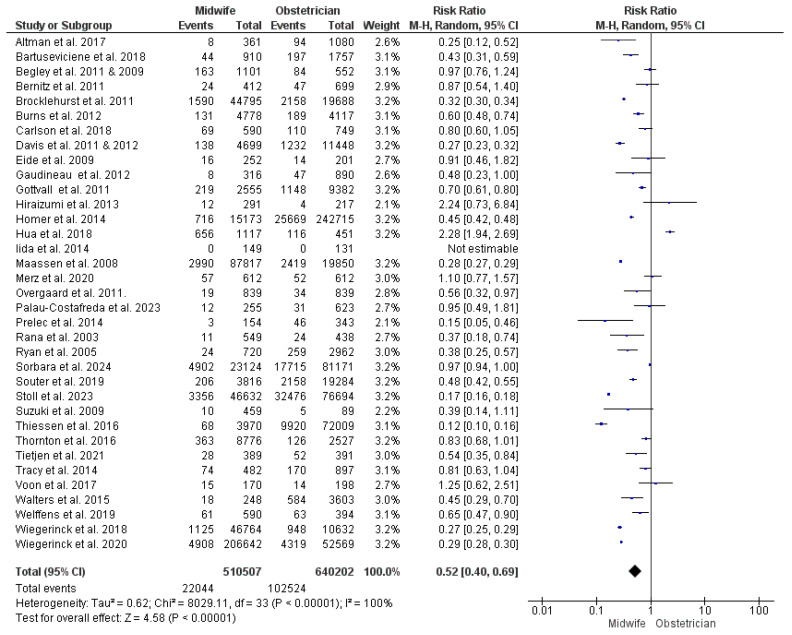
Forest plot of the analysis; unplanned cesarean section [22,23,24,27,28,29,30,35,36,37,39,43,44,45,47,48,49,50,51,52,53,54,55,56,57,59,60,61,62,63,65,66,67,68,69].

Thirty-five studies were included in this synthesis [22,23,24,27,28,29,30,34,35,36,37,38,39,43,44,45,47,48,49,50,51,52,53,54,55,56,57,58,59,60,61,62,63,65,66,67,68,69], with 1,150,709 women enrolled (510,507 received midwife-led care and 640,202 received obstetrician-led care). Our analysis significantly illustrated a lower risk of unplanned CS with midwife-led care (RR = 0.52; 95% CI [0.40, 0.69], *p* < 0.001). However, the findings across the studies showed heterogeneity (*p* < 0.001, I^2^ = 100%) *(*Figure 2).

##### Instrumental Vaginal Delivery

**Figure 3:**
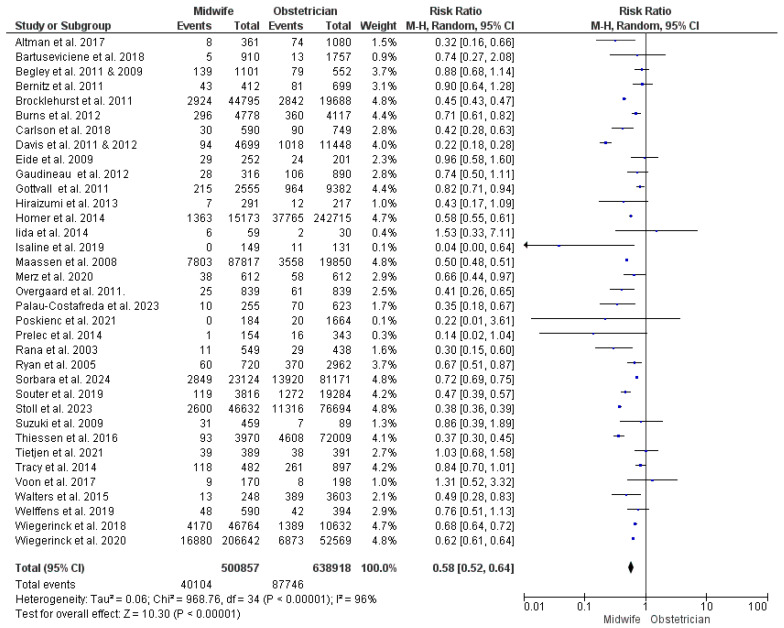
Forest plot of the analysis; instrumental vaginal delivery [22,23,24,27,28,29,30,35,36,37,39,43,44,45,47,48,49,50,51,52,53,54,55,56,57,59,60,61,62,63,65,66,67,68,69].

This synthesis was based upon results from 35 studies [22,23,24,26,27,28,29,30,31,34,35,36,38,39,43,44,47,48,49,50,51,52,53,54,55,56,57,58,59,60,61,62,63,65,66,67,68,69], with 1,139,775 women participating (294,215 received midwife-led care and 586,349 received obstetrician-led care). The risk of instrumental vaginal delivery was found to be significantly lower with midwife-led perinatal care (RR = 0.58; 95% CI [0.52, 0.64], *p* < 0.00001). Results showed heterogeneity between the studies included in this synthesis (*p* < 0.001, I^2^ = 96%) (Figure 3).

##### Epidural or Spinal Analgesia

**Figure 4:**
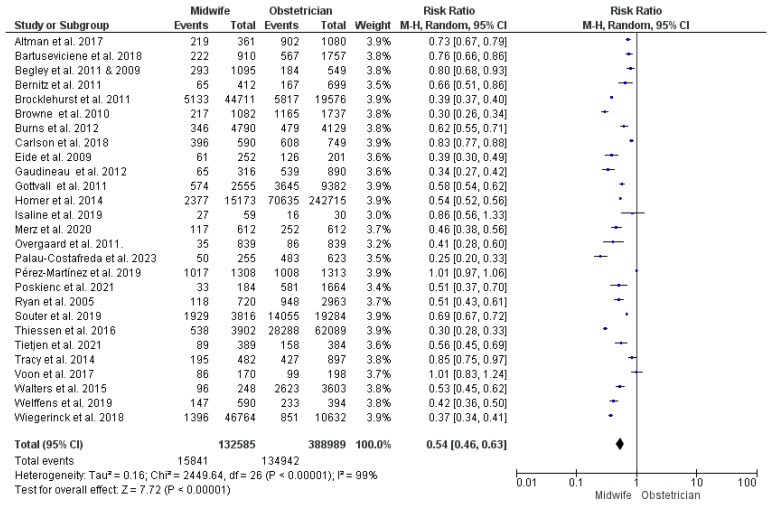
Forest plot of the analysis; epidural or spinal analgesia [22,23,24,27,28,29,30,35,36,37,39,43,44,45,47,48,49,50,51,52,53,54,55,56,57,59,60,61,62,63,65,66,67,68,69].

Twenty-seven studies contributed data for this synthesis [23,26,27,28,29,31,33,34,35,36,38,39,43,44,47,48,51,53,54,55,56,57,58,59,62,63,64,65,68], with 521,574 women included (132,585 received midwife-led care and 388,989 received obstetrician-led care). Midwife-led perinatal care was detected to carry a significantly lower risk of using epidural or spinal analgesia (RR = 0.54; 95% CI [0.46, 0.63], *p* < 0.00001). However, heterogeneity across the studies’ findings was significant (*p* < 0.001, I^2^ = 99%) (Figure 4).

#### PPH

**Figure 5:**
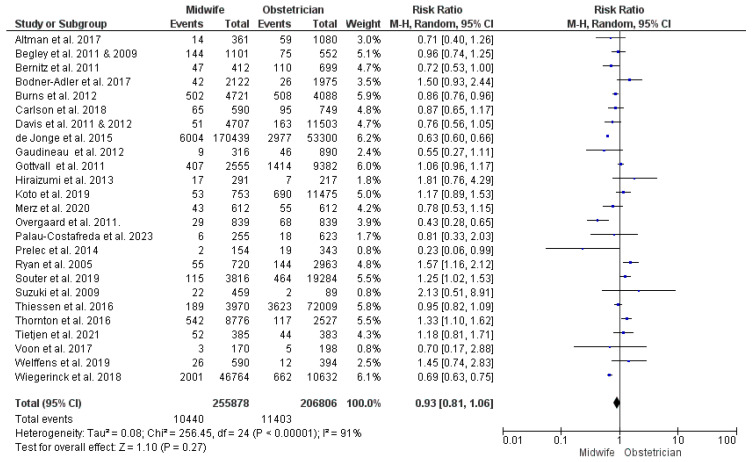
Forest plot of the analysis; postpartum hemorrhage [22,23,24,27,28,29,30,35,36,37,39,43,44,45,47,48,49,50,51,52,53,54,55,56,57,59,60,61,62,63,65,66,67,68,69].

This synthesis was conducted on data from 25 studies [23,27,28,29,32,34,36,38,39,40,41,43,44,45,46,50,52,53,54,55,56,57,58,60,61,62,63,66,68], with 462,684 women participating (255,878 received midwife-led care and 206,806 received obstetrician-led care). The risk of PPH did not differ significantly between the two models of perinatal care (RR = 0.93; 95% CI [0.81, 1.06], *p* = 0.27). The findings showed heterogeneity across the studies included in this synthesis (*p* < 0.001, I^2^ = 91%) (Figure 5).

##### APGAR Score < 7

**Figure 6:**
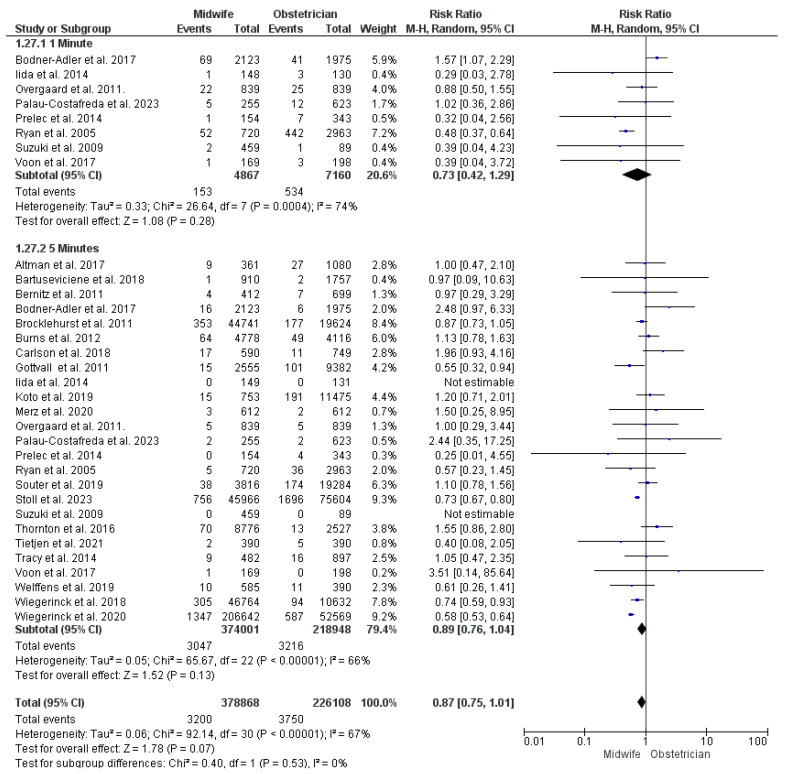
Forest plot of the analysis; APGAR score < 7 [22,23,24,27,28,29,30,35,36,37,39,43,44,45,47,48,49,50,51,52,53,54,55,56,57,59,60,61,62,63,65,66,67,68,69].

At one minute: Eight studies were included in the analysis of one-minute APGAR score < 7 [23,40,41,43,49,50,63,66,68], with 12,027 women participating (4867 received midwife-led care and 7160 received obstetrician-led care). No significant difference was detected between the two studied models, and the findings were heterogeneous (RR = 0.73; 95% CI [0.42, 1.29], *p* = 0.28), (*p* < 0.001, I^2^ = 74%) (Figure 6).

At five minutes: This synthesis was based upon 25 studies [23,24,28,29,30,32,34,35,36,38,39,40,41,43,44,45,49,50,51,53,57,58,59,62,63,66,68], with 592,949 women enrolled (374,001 received midwife-led care and 218,948 received obstetrician-led care). No significant difference was detected between midwife-led and obstetrician-led perinatal care models in the risk of five-minute APGAR score < 7, but the studies’ findings were heterogeneous (RR = 0.89; 95% CI [0.76, 1.04], *p* = 0.13), (*p* < 0.001, I^2^ = 66%) (Figure 6).

##### Intrapartum or Neonatal Mortality

**Figure 7:**
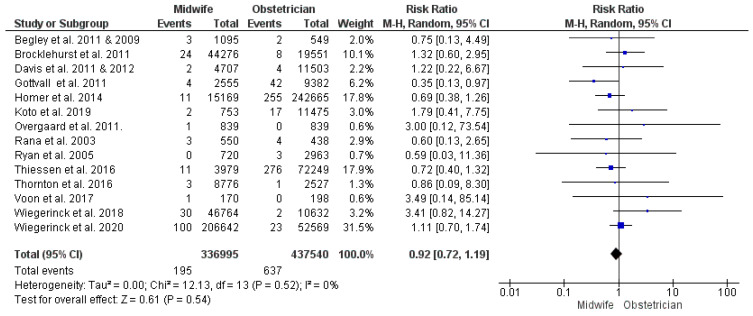
Forest plot of the analysis; intrapartum or neonatal mortality [35,39,43,45,47,48,49,50,51,53,54,55,56,57,59,60,61,62,63,65,66,67,68,69].

The intrapartum and neonatal mortality analysis was conducted on the findings of 14 studies [30,32,38,43,44,45,48,55,56,59,60,61,62,63,68,69], with 774,535 enrolled women (336,995 received midwife-led care and 437,540 received obstetrician-led care). The difference between midwife-led and obstetrician-led models of care in the risk of intrapartum or neonatal mortality was not statistically significant (RR = 0.92; 95% CI [0.72, 1.19], *p* = 0.54). The studies’ findings on this outcome were homogeneous (*p* = 0.52, I^2^ = 0%) (Figure 7).

#### 3.4.2. Secondary Outcomes

##### Birth Interventions

Lower risks of labor augmentation (*p* < 0.00), episiotomy (*p* < 0.001), and general anesthesia (*p* = 0.03) were noticed with midwife-led perinatal care. In addition, women who received midwife-led care had more physiological management of labor in the third stage (*p* < 0.001) and used more non-pharmacological acupuncture pain relief (*p* = 0.001) or no pain relief at all (*p* = 0.02). No significant difference was detected between midwife-led and obstetrician-led models of care in regard to the risk of CS because of suspected fetal distress or non-progressive labor, NO2 anesthesia, pudendal nerve block, narcotics use, or hydrotherapy pain relief (Table 3), (Figures S3–S13).

##### Maternal Outcomes

The risk of various degrees of perineal tear was not significantly different between the two studied models of perinatal care, except for the risk of vaginal tear, which was higher with the midwife-led model (*p* = 0.01). Women receiving midwife-led perinatal care had lower risks of manual removal of the placenta (*p* < 0.001) and blood transfusion (*p* = 0.03). Moreover, these women had a lower risk of maternal infection or fever (*p* = 0.01) and maternal ICU admission (*p* < 0.01), besides shorter hospital stays (*p* = 0.02). The risk of severe maternal morbidity and the duration of labor did not vary significantly between the midwife-led and obstetrician-led models of care (Table 3), (Figures S14–S24).

##### Neonatal Outcomes

Mean APGAR score at one minute was higher for neonates born under the midwife-led care model (*p* = 0.009). However, the five-minute mean score did not differ significantly between the two models. Midwife-led perinatal care was superior to obstetrician-led care in regard to the risk of umbilical cord arterial pH < 7.1 (*p* = 0.04), asphyxia (*p* = 0.03), transfer to specialist neonatal care (*p* = 0.03), and NICU admission (*p* = 0.01). Mean umbilical cord arterial pH and the risks of needing resuscitation or ventilation, meconium-stained liquor, and shoulder dystocia did not differ significantly between midwife-led and obstetrician-led models of care. Midwife-led care showed superiority with regard to breastfeeding initiation (*p* < 0.001) (Table 3), (Figures S25–S35).

### 3.5. Publication Bias

There appears to be some evidence of publication bias in four outcomes: breastfeeding initiation, episiotomy, instrumental vaginal delivery, and NICU admission. The funnel plots revealed asymmetry, suggesting that smaller studies with negative or non-significant results may be underrepresented. This asymmetry highlights the need for caution when interpreting the results, as the observed effects might be influenced by the selective publication of studies with positive findings (Figures S36–S39). However, the rest of the outcomes did not show evidence of publication bias (Figures S40–S49).

### 3.6. Qualitative Synthesis

#### 3.6.1. Women’s Satisfaction with Care

Bernitz et al. and Begley et al. detected higher satisfaction levels among low-risk women randomized to midwife-led perinatal care. However, intrapartum transfer of care was noticed to influence women’s satisfaction negatively [56,58]. The high satisfaction levels were predominantly noted with the continuity model of midwife care [37,49].

#### 3.6.2. Studies Not Included in the Analysis

Martin-Arribas et al., 2022: The study showed that women under midwife-led care had lower rates of cesarean section, instrumental vaginal delivery, epidural analgesia, and augmentation of labor (*p* < 0.001 for all outcomes). Women receiving midwife-led care had lower rates of third or fourth-degree perineal tear (*p* < 0.001), active management of the third stage of labor (*p* < 0.001), and ICU admissions (*p* = 0.01). Neonates born under this model of care had lower rates of APGAR score < 7 at five minutes (*p* = 0.01) and neonatal resuscitation (*p* < 0.001) and higher rates of breastfeeding initiation (*p* < 0.001) [25].

Schroeder et al., 2017: Categorical data reported in this study were not presented as events and total to allow for analysis. Cesarean section was less frequent with midwife-led care (*p* = 0.003). However, the rates of instrumental vaginal delivery, labor augmentation, and manual removal of the placenta did not vary significantly between the two models of care. Using birth pools for pain was more frequent with midwife-led perinatal care (*p* < 0.001), whereas using pethidine was more frequent with obstetrician-led care (*p* = 0.02) [42].

## 4. Discussion

This systematic review summarized the findings of 44 studies comparing midwife-led with obstetrician-led perinatal care for women with low-risk pregnancies. Nearly 1.4 million women (*n* = 1,397,320) were enrolled in studies conducted in 21 different countries. Our synthesis concluded that the midwife-led model of care carried a lower risk of birth interventions (namely, unplanned CS, instrumental vaginal delivery, augmentation of labor, epidural or spinal analgesia, general anesthesia, and episiotomy). More women in the midwife-led care group received physiological management of the third stage of labor. They did not use any pain relief intervention or used non-pharmacological acupuncture pain relief. This reduction of childbirth interventions by midwives was not noticed to adversely influence maternal and neonatal outcomes. Instead, some maternal and neonatal outcomes were more favorable with the midwife-led perinatal care. The risks of PPH, perineal injuries, and severe maternal morbidity did not differ significantly between the two models of management. However, women who received midwife-led care had shorter hospital stays and lower risks of infection or fever, manual removal of the placenta, blood transfusion, and ICU admission. Conversely, these women had a higher risk of vaginal tears compared with women delivering under obstetrician-led care. The duration of labor did not vary significantly between the two models of care. Regarding neonatal outcomes, the risks of having an APGAR score < 7 (at one minute and five minutes), meconium-stained liquor, shoulder dystocia, needing resuscitation or ventilation, and intrapartum or neonatal mortality did not differ between the two models of care. Yet, newborns delivered under midwife-led care had lower risks of acidosis (umbilical cord arterial pH < 7.1), asphyxia, transfer to specialist care, and NICU admission. These neonates were also more likely to start breastfeeding. Our findings indicate that midwife-led care is associated with fewer medical interventions and favorable maternal and neonatal outcomes in most cases. This supports the potential benefits of midwife-led care for low-risk pregnancies. However, it is important to clarify that our analysis focused on the incidence rates of interventions such as unplanned cesarean delivery and epidural/spinal analgesia, rather than on who performed these interventions. In all the included studies, cesarean deliveries were performed by doctors. Our analysis primarily centered on prenatal care and the overall management provided by midwives versus obstetricians. By focusing on incidence rates, we aimed to provide a comparative analysis of the frequency of these interventions in different care models, irrespective of the practitioner performing them. This approach allows us to highlight the differences in care practices and outcomes between midwife-led and obstetrician-led models, while acknowledging that certain interventions require the involvement of an obstetrician. This distinction is crucial for understanding the scope and limitations of midwife-led care and underscores the importance of integrated care models that facilitate collaboration between midwives and obstetricians to ensure comprehensive care for all patients.

The observed differences in outcomes between midwife-led and obstetrician-led care can be attributed to several factors. Midwife-led care often emphasizes a more holistic and less interventionist approach to childbirth, focusing on the natural birthing process and providing continuous support to the mother. This model of care is associated with lower rates of medical interventions such as labor augmentation, epidural analgesia, and cesarean sections. Midwives are trained to manage normal pregnancies and deliveries, and their approach is often centered on promoting normalcy and minimizing unnecessary interventions. This philosophy aligns with the preferences of many women who seek a more natural childbirth experience. In contrast, obstetrician-led care is typically more medicalized, with a greater emphasis on the use of technology and interventions to manage potential complications. Obstetricians are trained to handle high-risk pregnancies and are more likely to intervene proactively to prevent adverse outcomes. This approach can lead to higher rates of interventions, even in low-risk pregnancies, as a precautionary measure. The differences in training, philosophy, and approach between midwives and obstetricians contribute to the variations in outcomes observed in our study. Additionally, the continuity of care provided by midwives, who often build a strong rapport with their patients, can lead to better communication, increased trust, and a more personalized birthing experience. This continuity and personalized care can positively impact maternal satisfaction and outcomes. On the other hand, obstetrician-led care may involve multiple providers, which can sometimes lead to fragmented care and less personalized attention.

A previous meta-analysis published by Sandall et al. in 2016 compared the hospital-based midwife-led continuity model of care with other models of care during pregnancy and childbirth [70]. This meta-analysis included 15 RCTs with 17,674 women enrolled. Our meta-analysis was not limited to continuity midwife-led care, as we included all midwife-led models of care. Another review of reviews was published in 2012 by Sutcliffe et al. summarizing the findings of 27 studies included in three meta-analyses (Hatem et al. 2008, Villar et al. 2001, and Brown and Grimes 1995) [71,72,73,74]. Sutcliffe et al. compared midwife-led maternity care with physician-led care for low-risk pregnancies. In this review, we focused our comparison on obstetrician-led perinatal care to allow for drawing specific recommendations for implementation in practice. Overall, the findings of Sandall et al. and Sutcliffe et al. were consistent with ours. They concluded that midwife-led perinatal care applied fewer interventions during childbirth without increasing the risk of maternal bleeding or infections and without compromising neonatal outcomes. All previous reviews agreed with us on the reduced risk of instrumental vaginal delivery, episiotomy, and epidural analgesia with midwife-led care. The reviews also found an insignificant difference in the risks of perineal injuries and intrapartum or neonatal mortality between the two models of care [70,71]. The findings of Sutcliffe et al. were consistent with ours regarding the risks of PPH and five-minute Apgar score < 7 [71]. We did not detect a difference in the duration of labor between the two models of care, a finding consistent with Sutcliffe et al. as well [71]. Unlike previous reviews, our analysis revealed a lower risk of cesarean section with midwife-led care [70,71]. Our findings on the risks of labor augmentation, manual removal of the placenta, and NICU admission are also not consistent with those of Sutcliffe et al. [71]. We assume that these differences emerge from our comparison focused on the tertiary obstetrician-led level of care rather than including the primary family physician-led level of care. Tertiary obstetrician-led care has accessibility to the most specialized professionals and advanced care units, which makes interventions like CSs and admissions to NICU more reachable. Although family physicians are doctors, they operate at a primary healthcare level and are not the professionals who perform advanced obstetric interventions.

The American College of Obstetricians and Gynecologists has emphasized the importance of reducing unnecessary interventions during childbirth [75]. Several countries have also advocated for this goal [76]. The obstetrician-led model of care uses medications and obstetric interventions aiming to reduce maternal complications (mainly bleeding and infections) and neonatal adverse outcomes [77,78]. On the contrary, midwives support the normal biological demedicalized process of birth [14]. Our review showed that midwife-led care reduced childbirth interventions without increasing the risk of maternal bleeding. Instead, midwife-led care resulted in a lower risk of maternal infection and more favorable neonatal outcomes. Moreover, midwife-led care has psychological superiority over obstetrician-led care. Previous studies noticed a lower level of maternal anxiety, an accentuated sense of control, and enhanced levels of satisfaction with midwife-led perinatal care [9,13,71,79].

Our findings support shifting low-risk women to give birth under midwives’ care. This shift would allow obstetricians to devote their time to higher-risk women requiring special care for complications. Such a shift was questionable before, particularly when concerns were raised about the quality of midwife-led, un-totally supervised care [80,81]. Our review summarized the studies’ findings, providing answers to these concerns. Shifting low-risk women to midwife-led perinatal care would also minimize the human resources gap in low-income countries and remote areas where obstetricians are not always accessible [82]. This inaccessibility to emergency obstetric services and skilled birth attendants is enormously responsible for high rates of maternal mortality and morbidity [83]. Midwives’ easy training, wide accessibility, and low salary make them ideal practitioners at the primary care level [84]. They can provide high-quality perinatal care for low-risk women besides primary emergency obstetric care and facilitate smooth transfer of complicated, risky cases [81,85,86,87]. A shorter duration and only one-third of the cost of training a physician are sufficient to train a midwife. Furthermore, midwives’ salaries are less than half those of physicians. Previous studies have proved that midwife-led care is a cost-effective alternative to physician-led care [88].

Most institutions define a low-risk pregnancy as an uncomplicated singleton pregnancy in a cephalic-vertex presentation in a healthy woman. However, the criteria for “uncomplicated pregnancy” and “healthy woman” encompass significant variability [77]. Setting unified agreed-upon criteria for low-risk pregnancy is required to identify women eligible for childbirth under midwife-led care. Planning birth should take into consideration the pregnancy risk status and women’s preferences. However, the birth plan could be adjusted at multiple points, from the anomaly scan in the second trimester, the growth scan in the third trimester, late antenatal visits, by the onset of labor, or even during labor. Thus, counseling women about the birth plan and its flexibility is better conveyed in more than one session. Studies reported the highest satisfaction levels when women were involved in planning childbirth [89,90,91].

In this review, data were insufficient to perform a subgroup analysis by the place of birth. However, several previous reviews have revealed an insignificant difference between hospital and community births. Some maternal and neonatal outcomes have even favored community birth [9,79,92,93,94,95,96]. Transfer of care is a substantial part of any primary healthcare service. It should be expected and planned in advance to avoid the three delays causing maternal mortality. Pre-specified guidelines for the criteria on who to transfer should be set by the regulatory bodies. Furthermore, clear, respectful communication between perinatal care providers and birth settings is needed. For births occurring in FMUs and homes, suitable, readily available means of transportation are also required. These three requisites mentioned above make a smoothly articulated transfer system. In low-income countries, the infrastructure is poor for timely transportation from community to hospital. Additionally, communication between community midwives and hospitals is not optimally regulated. Thus, the transfer system is questionable in such settings. The situation in low-resource countries is understated in this review due to insufficient data. Further research from these countries is needed.

It was in the 1990s when a call for reducing unnecessary childbirth interventions emerged. In 1996, the World Health Organization issued a statement about assigning midwives to perinatal care during normal pregnancy and childbirth [76]. After that, regulatory boards in several countries responded to this call. It was around the year 2000 when midwife-led care was officially regulated in several countries [97,98,99]. Therefore, in this review, we included studies published after 2000. This review is strengthened by the large number of included studies representing various nations and health systems. Furthermore, the enormously large number of participants who were enrolled empowered the included studies. It allowed this review to analyze rare outcomes such as the risks of intrapartum or neonatal mortality and third- or fourth-degree perineal tear. Yet, maternal mortality was not reported or did not occur in most of the included studies. Although the gold standard in providing trusted evidence is the RCT, childbirth is a decision that is not generally acceptable to be taken randomly. Conducting an RCT on such a topic is not typically feasible [100,101]. Due to the limited number of published RCTs, we included observational studies in this review. However, the confounding issue was adjusted for in the analysis of most of the observational studies included. Another limitation of this review is the issue of blinding study participants, staff, and outcomes assessors. All the included studies were open-label. Blinding the participants might not be feasible due to the setting in RCTs and their choice in observational studies. Additionally, blinding the study personnel is not possible. Future studies might consider blinding outcomes assessors when feasible, as some outcomes might be influenced, such as evaluating perineal injuries. The same issue was reported in the previous review by Sandall et al. [70]. Another limitation faced by previous reviews is the synthesis of women’s satisfaction [70]. We recommend developing a tool for assessing women’s satisfaction during childbirth that can be used consistently. Such a tool would allow for quantitative synthesis and comparison. Due to the variety in the represented health systems, birth settings, definition of low-risk pregnancy, and studies’ design, heterogeneity across the included studies was significant in most studied outcomes. Yet again, this issue was reported by several previous reviews [70,79,102]. Moreover, the substantial heterogeneity observed in our meta-analysis, indicated by an I² value of 100%, highlights significant variability among the included studies. This high level of heterogeneity can be attributed to several factors, including differences in study design, population characteristics, interventions, and outcome measures. To address this, we conducted subgroup analyses and sensitivity analyses to explore potential sources of heterogeneity. These analyses revealed that variations in study settings, sample sizes, and methodological approaches contributed to the observed heterogeneity. Additionally, we employed a random-effects model to account for the variability among studies, which provides more conservative estimates and acknowledges the diversity of the included studies. Despite these efforts, the high heterogeneity underscores the need for cautious interpretation of the results and suggests that further research is necessary to understand the underlying factors contributing to these variations.

## 5. Conclusions

Midwife-led care has resulted in a lower risk of birth interventions without increasing the risk of maternal infection, perineal injuries, or bleeding and without compromising neonatal outcomes. Midwives are cost-effective and widely available alternatives to offer primary healthcare services and allow obstetricians to dedicate their efforts to more complex cases. We recommend assigning low-risk women to midwife-led care in nations with an infrastructure system that allows for smooth transfer when needed. Further research from low-resource countries is required to reflect their situation.

## Figures and Tables

**Table 1:** Description of the methodology of the included studies.

Study ID	Study Design	Country	Sample Size	Eligibility Criteria	MW-Led Care Place of Birth	OB-Led Care Place of Birth	Outcomes Relevant to This Review	Antenatal Care
Sorbara et al., 2024 [22]	Retrospective cohort	Canada	104,995 women (23,124 for MW-led care and 81,871 for OB-led care)	Nulliparous women aged 11 to 50 years with a singleton pregnancy beyond 34 weeks of gestation. Women were excluded if they had a stillbirth, previous CS, previous preterm labor, history of pre-eclampsia, or missing BMI.	AMU	Hospital	Unplanned CS, instrumental vaginal delivery, 3rd/4th degree perineal tear, and episiotomy.	-
Palau-Costafreda et al., 2023 [23]	Retrospective cohort	Spain	878 women (255 for MW-led care and 623 for OB-led care)	Under 40-year-old women with singleton pregnancy between 37 and 42 weeks of gestation, cephalic presentation, parity <4, pre-pregnancy BMI ≤30, and Hb level ≥10 g/dL at labor. Women were excluded if they had recurrent or heavy vaginal bleeding, oligohydramnios/polyhydramnios, placenta Previa, pre-eclampsia, fetal major anomaly, estimated fetal weight ≥4.5 kg, gestational diabetes requiring insulin, hepatitis B or C or HIV infection, active genital herpes, active Rubella, Syphilis, Toxoplasma, or Cytomegalovirus infection with evidence of fetal infection, meconium-stained amniotic fluid or PROM, uncontrolled medical condition, previous uterine surgery (including CS), or a history of PPH, retained placenta, 4th-degree perineal laceration, or shoulder dystocia.	AMU	Hospital	Unplanned CS, instrumental vaginal delivery, epidural/spinal analgesia, general anesthesia, 1st/2nd-degree perineal tear, 3rd/4th degree perineal tear, intact perineum, episiotomy, PPH, manual removal of placenta, APGAR score < 7 at one minute, and APGAR score < 7 at five minutes.	-
Stoll et al., 2023 [24]	Retrospective cohort	Canada	123,326 women (46,632 for MW-led care and 76,694 for OB-led care)	Low-risk pregnancies as judged by a scoring system derived from the International Classification of Diseases-10th Revision.	Home or AMU	Hospital	Unplanned CS, instrumental vaginal delivery, 3rd/4th degree perineal tear, APGAR score < 7 at five minutes, and breastfeeding initiation.	-
Martin-Arribas et al., 2022 [25]	Cross-sectional	Spain	11,537 women (10,844 for MW-led care and 693 for OB-led care)	Eighteen to 40-year-old women with singleton pregnancy between 37 and 42 weeks of gestation, cephalic presentation, having low- or moderate-risk pregnancy as judged by Catalonia (Spain) guidelines of antenatal care.	AMU	Hospital	Unplanned CS, instrumental vaginal delivery, epidural/spinal analgesia, augmentation of labor, 3rd/4th-degree perineal tear, physiological management of the third stage of labor, maternal ICU admission, APGAR score < 7 at five minutes, need for resuscitation, and breastfeeding initiation.	-
Poskienc et al., 2021 [26]	Case-control	Lithuania	1848 women (184 for MW-led care and 1664 for OB-led care)	Women having an uncomplicated singleton term pregnancy with a well-developed fetus planned for vaginal delivery. Participants should be healthy before and during pregnancy.	FMU or AMU	Hospital	Instrumental vaginal delivery, epidural/spinal analgesia, NO2 analgesia, no pain relief, 1st/2nd-degree perineal tear, 3rd/4th degree perineal tear, intact perineum, episiotomy, manual removal of placenta, hospital stay duration, one minute APGAR score, five-minute APGAR score, and breastfeeding initiation.	-
Tietjen et al., 2021 [27]	Prospective cohort	Germany	782 women (391 for MW-led care and 391 for OB-led care)	Healthy women with a singleton term cephalic pregnancy. Women with medical disorders, complicated pregnancies (e.g., pre-eclampsia, fetal abnormalities, or oligohydramnios), or abnormal obstetric history (e.g., previous CS) were excluded.	AMU	Hospital	Unplanned CS, instrumental vaginal delivery, epidural/spinal analgesia, 1st/2nd-degree perineal tear, 3rd/4th degree perineal tear, intact perineum, episiotomy, PPH, duration of labor, duration of hospital stay, APGAR score < 7 at five minutes, one-minute APGAR score, five-minute APGAR score, umbilical cord arterial pH < 7.1, umbilical cord arterial pH, and transfer to specialist neonatal care.	-
Merz et al., 2020 [28]	Retrospective cohort	Germany	1224 women (612 for MW-led care and 612 for OB-led care)	Women with a term singleton cephalic pregnancy were included. Women having a BMI of > 35 kg/m^2^, medical condition, complicated pregnancy (e.g., gestational diabetes requiring insulin, pre-eclampsia/pregnancy-induced hypertension, oligohydramnios/polyhydramnios), previous history of obstetric complications (e.g., PPH, CS, shoulder dystocia) were excluded.	AMU	Hospital	Unplanned CS, instrumental vaginal delivery, epidural/spinal analgesia, 1st/2nd-degree perineal tear, 3rd/4th degree perineal tear, intact perineum, episiotomy, PPH, APGAR score < 7 at five minutes, umbilical cord arterial pH < 7.1, and transfer to specialist neonatal care.	-
Welffens et al., 2019 [29]	Retrospective cohort	Belgium	984 women (590 for MW-led care and 394 for OB-led care)	Women ≥ 18 and ≤40-year-old having a singleton cephalic pregnancy with normal fetal weight and gestational age between 37 and 42 weeks at labor onset. Women were excluded if they had medical disorders, parity more than six, pre-pregnancy BMI ≥ 35 or <18 (kg/m^2^), complicated pregnancy (pre-eclampsia, eclampsia, induction of labor, oligohydramnios/polyhydramnios, placental abnormality, or major fetal anomaly), abnormal obstetric/gynecological history (severe pre-eclampsia, idiopathic IUFD, neonatal septicemia of group B streptococcus, previous myomectomy or hystrotomy), gestational diabetes with suspected fetal macrosomia or poor glycemic control, Hb level < 9 g/dL at 36 weeks of gestation, substance abuse, gestational age ≥ 32 weeks at start of prenatal care, beginner communication level in French, or poor motivation for natural birth.	AMU	Hospital	Unplanned CS, instrumental vaginal delivery, epidural/spinal analgesia, 1st/2nd-degree perineal tear, 3rd/4th degree perineal tear, episiotomy, PPH, manual removal of placenta, APGAR score < 7 at five minutes, and transfer to specialist neonatal care.	Part of the study
Wiegerinck et al., 2020 [30]	Retrospective cohort	The Netherlands	259,211 women (206,642 for MW-led care and 52,569 for OB-led care)	Women with singleton term pregnancies in a cephalic presentation planned for vaginal delivery. Exclusion criteria were: DM, hypertension, induction of labor, post-term pregnancy, antepartum hemorrhage, antenatal stillbirth, fetal anomaly, PROM, or previous CS.	Home, FMU, or AMU	Hospital	Unplanned CS, instrumental vaginal delivery, APGAR score < 7 at five minutes, and intrapartum or neonatal mortality.	-
Isaline et al., 2019 [31]	Retrospective cohort	Belgium	89 women (59 for MW-led care and 30 for OB-led care)	18- to 40-year-old healthy women with uncomplicated pregnancy and spontaneous labor. Women with no Belgian social identification number were excluded.	AMU	Hospital	Instrumental vaginal delivery, epidural/spinal analgesia, 1st/2nd-degree perineal tear, intact perineum, episiotomy, duration of hospital stay, NICU admission, and breastfeeding initiation.	-
Koto et al., 2019 [32]	Retrospective cohort	Canada	12,228 women (753 for MW-led care and 11,475 for OB-led care)	Women with singleton cephalic pregnancy planned for vaginal delivery between 37 and 41 weeks of gestation. Women were excluded if they had pre-existing or pregnancy-related DM or hypertension, medical conditions (e.g., blood dyscariasis, heart disease, endocrine disorder), complicated pregnancy (e.g., major fetal congenital anomaly, low birth weight (<2.5 kg), or placenta previa/accreta/increta/percreta), or CS within two years.	AMU	Hospital	PPH, blood transfusion, maternal ICU admission, maternal infection/fever, APGAR score < 7 at five minutes, NICU admission, breastfeeding initiation, and intrapartum or neonatal mortality.	-
Pérez-Martínez et al., 2019 [33]	Retrospective quasi-experimental	Spain	2621 women (1308 for MW-led care and 1313 for OB-led care)	Low-risk postpartum women	AMU	Hospital	Epidural/spinal analgesia, general anesthesia, local analgesia/pudendal block, no pain relief, 1st/2nd-degree perineal tear, 3rd/4th degree perineal tear, intact perineum, episiotomy, NICU admission, and breastfeeding initiation.	-
Souter et al., 2019 [34]	Retrospective cohort	The United States	23,100 women (3816 for MW-led care and 19,284 for OB-led care)	Women with singleton term cephalic pregnancy planned for vaginal delivery. Women were excluded if they had ≥43 weeks pregnancy, age ≥45 years, BMI ≥ 40 kg/m^2^, pre-existing or pregnancy-related DM or hypertension, complicated pregnancy (any fetal anomaly, antepartum stillbirth, induction of labor unless for post-date), abnormal obstetric history (previous CS, stillbirth, cholestasis), inadequate antenatal care, smoking, alcohol use, or illegal substances use.	AMU	Hospital	Unplanned CS, instrumental vaginal delivery, epidural/spinal analgesia, augmentation of labor, 3rd/4th-degree perineal tear, episiotomy, PPH, blood transfusion, severe maternal morbidity, APGAR score < 7 at five minutes, need for neonatal resuscitation, and shoulder dystocia.	-
Bartuseviciene et al., 2018 [35]	Retrospective cohort	Lithuania	2667 women (910 for MW-led care and 1757 for OB-led care)	Women with singleton cephalic term uncomplicated pregnancy planned for vaginal delivery presenting in spontaneous labor. Women were excluded if they had medical disorders, BMI ≥ 30 kg/m^2^, age >40 years, or pregnancy beyond 42 weeks.	AMU	Hospital	Unplanned CS, instrumental vaginal delivery, epidural/spinal analgesia, augmentation of labor, 1st/2nd-degree perineal tear, 3rd/4th-degree perineal tear, episiotomy, duration of labor, APGAR score < 7 at five minutes, and five-minute APGAR score.	-
Carlson et al., 2018 [36]	Retrospective cohort	The United States	1339 women (590 for MW-led care and 749 for OB-led care)	Nulliparous 18- to 40-year-old women with singleton term cephalic-vertex pregnancy planned for vaginal delivery who presented in spontaneous labor. Women were excluded for complicated pregnancy (IUFD, IUGR, post-term pregnancy, fetal major anomaly, PROM, oligohydramnios/anhydramnios, or pre-eclampsia), pre-existing or pregnancy-related DM or hypertension, severe co-morbidity (e.g., major cardiac disease, cancer), self-selecting private family practice or midwifery care, or missing data to calculate BMI.	AMU	Hospital	Unplanned CS, CS of suspected fetal distress, CS of non-progressive labor, instrumental vaginal delivery, epidural/spinal analgesia, augmentation of labor, 3rd/4th-degree perineal tear, PPH, maternal infection/fever, APGAR score < 7 at five minutes, NICU admission, need for ventilation, and shoulder dystocia.	-
Hua et al., 2018 [37]	Prospective cohort	China	1568 women (451 for MW-led care and 1117 for OB-led care)	Nulliparous healthy women with singleton uncomplicated pregnancy at 29 to 30 weeks of gestation at recruitment. Women were excluded for having fetal anomalies, severe personal or family medical or psychiatric history, or inability to consent.	AMU	Hospital	Unplanned CS and breastfeeding initiation.	After 30 weeks
Wiegerinck et al., 2018 [38]	Retrospective cohort	The Netherlands	57,396 women (46,764 for MW-led care and 10,632 for OB-led care)	Women with a term pregnancy in vertex presentation planned for vaginal delivery. Women were excluded for complicated pregnancy (post-term, antenatal stillbirth, major fetal anomaly, PROM, antepartum hemorrhage, or induction of labor), pregnancy-related DM, hypertension, or previous CS.	Home or AMU	Hospital	Unplanned CS, CS of suspected fetal distress, CS of non-progressive labor, instrumental vaginal delivery, epidural/spinal analgesia, 3rd/4th-degree perineal tear, episiotomy, PPH, manual removal of placenta, APGAR score < 7 at five minutes, NICU admission, and intrapartum or neonatal mortality.	-
Altman et al., 2017 [39]	Retrospective cohort	The United States	1441 women (361 for MW-led care and 1080 for OB-led care)	Women with singleton term pregnancy in cephalic-vertex presentation. Women were excluded if they had complicated pregnancy (post-term, placenta previa, pre-eclampsia, or fetal anomalies), pre-existing or pregnancy-related DM or hypertension, seizure disorders, hypercoagulability, or previous CS.	AMU	Hospital	Unplanned CS, instrumental vaginal delivery, epidural/spinal analgesia, augmentation of labor, 3rd/4th-degree perineal tear, episiotomy, PPH, blood transfusion, duration of hospital stay, maternal infection/fever, APGAR score < 7 at five minutes, and NICU admission.	-
Bodner-Adler et al., 2017 and 2004 [40,41]	Retrospective cohort	Austria	4098 women (2123 for MW-led care and 1975 for OB-led care)	Healthy women with singleton term pregnancy in cephalic presentation. Women were excluded if they had DM, hypertension, complicated pregnancy (PROM, fetal anomaly, macrosomia, or retardation, or fetal/placental disease), history of CS or IUFD, or desire for epidural analgesia.	AMU	Hospital	Augmentation of labor, hydrotherapy pain relief, no pain relief, 1st/2nd-degree perineal tear, 3rd/4th-degree perineal tear, episiotomy, vaginal tear, labial tear, PPH, maternal infection/fever, APGAR score < 7 at one minute, APGAR score < 7 at five minutes, and umbilical cord arterial pH < 7.1.	-
Schroeder et al., 2017 [42]	Retrospective cohort	The United Kingdom	331 women (167 for MW-led care and 164 for OB-led care)	Healthy women with uncomplicated singleton term pregnancy in cephalic presentation presenting in spontaneous labor. Post-term pregnancies and women experiencing PROM were excluded.	FMU	Hospital	Unplanned CS, instrumental vaginal delivery, augmentation of labor, general anesthesia, narcotics use, hydrotherapy pain relief, and manual removal of the placenta.	-
Voon et al., 2017 [43]	Retrospective cohort	Singapore	368 women (170 for MW-led care and 198 for OB-led care)	Women aged 21 to 45 years with singleton term uncomplicated pregnancy in cephalic-vertex presentation planned for vaginal delivery. Women were excluded if they had pre-existing or pregnancy-related DM or hypertension, parity > 5, pregnancy complication (pre-eclampsia, eclampsia, antepartum hemorrhage, PROM, Rhesus isoimmunization in second or subsequent pregnancy, IUGR, oligohydramnios/polydramnios, fetal anomaly requiring specialized care, or in vitro fertilization), medical disorder (thyroid disease, renal disease, cardiac disease, neurological disease, neurosurgery, autoimmune disease, anemia, thalassemia, asthma, Guillain–Barre syndrome, hepatitis B infection, urinary tract infection), abnormal obstetric or gynecological history (sexually transmitted disease, CS or other uterine surgery, septate uterus, fibroids/cervical polyps > 5 cm, stillbirth or neonatal death, or recurrent abortion), psychological disorder, high BMI, or height < 152 cm.	AMU	Hospital	Unplanned CS, instrumental vaginal delivery, epidural/spinal analgesia, augmentation of labor, NO2 analgesia, narcotics use, no pain relief, intact perineum, 1st/2nd-degree perineal tear, 3rd/4th-degree perineal tear, episiotomy, PPH, duration of labor, duration of hospital stay, APGAR score < 7 at one minute, APGAR score < 7 at five minutes, NICU admission, breastfeeding initiation, and intrapartum or neonatal mortality.	After 28 to 32 weeks
Thiessen et al., 2016 [44]	Retrospective cohort	Canada	76,228 women (3979 for MW-led care and 72,249 for OB-led care)	Healthy women with uncomplicated singleton pregnancy. Women were excluded if they had complicated pregnancy (placenta previa, abruptio placentae, pregnancy-induced hypertension, or IUGR) or medical complications (e.g., cardiac disease or diabetes)	Home or AMU	Hospital	Unplanned CS, instrumental vaginal delivery, epidural/spinal analgesia, 1st/2nd-degree perineal tear, 3rd/4th-degree perineal tear, episiotomy, PPH, five-minute APGAR score, NICU admission, need for resuscitation, and intrapartum or neonatal mortality.	-
Thornton et al., 2016 [45]	Retrospective cohort	The United States	11,303 women (8776 for MW-led care and 2527 for OB-led care)	Women with term singleton pregnancy in vertex presentation and fetus weight between 2.5 and 6 kg, presenting in spontaneous labor. Women were excluded if they had pre-existing or pregnancy-related DM or hypertension, complicated pregnancy (fetal anomaly, PROM, placenta previa, placental abruption, pre-eclampsia, infection, or nonreassuring antepartum fetal testing), medical disorder (renal disease, history of seizure, asthma, or anemia), previous CS, sexual or physical abuse, history of psychiatric disorder, tobacco/substance abuse, or BMI < 20 or >27.	FMU	Hospital	Unplanned CS, PPH, APGAR score < 7 at five minutes, meconium-stained fluid, need for ventilation, breastfeeding initiation, and intrapartum or neonatal mortality.	-
De Jonge et al., 2015 [46]	Retrospective cohort	The Netherlands	223,730 women (170,430 for MW-led care and 53,300 for OB-led care)	Women with a singleton term pregnancy in cephalic presentation admitted in spontaneous labor. Women were excluded if they had a post-term pregnancy or a history of CS.	Home or AMU	Hospital	Augmentation of labor, PPH, manual removal of placenta, severe maternal morbidity, and maternal ICU admission.	-
Walters et al., 2015 [47]	Retrospective cohort	Canada	3851 women (248 for MW-led care and 3603 for OB-led care)	Women with singleton term pregnancy. Women were excluded if they had pre-existing or pregnancy-related DM or hypertension, complicated pregnancy (placenta previa, placental abruption, or malpresentation), previous CS, or previous stillbirth.	AMU	Hospital	Unplanned CS, instrumental vaginal delivery, epidural/spinal analgesia, and NICU admission.	-
Homer et al., 2014 [48]	Retrospective cohort	Australia	244,161 women (1225 for MW-led care and 242,936 for OB-led care)	Women with singleton term pregnancy in a cephalic presentation planned for vaginal delivery who presented in spontaneous labor. Women were excluded if they had no antenatal care, delivered before arrival, previous CS, and fetal congenital anomaly.	Home or FMU	Hospital	Unplanned CS, instrumental vaginal delivery, epidural/spinal analgesia, augmentation of labor, general anesthesia, 3rd/4th-degree perineal tear, episiotomy, and intrapartum or neonatal mortality.	-
Iida et al., 2014 [49]	Retrospective cohort	Japan	280 women (149 for MW-led care and 131 for OB-led care)	Women with singleton term pregnancy who received antenatal care and were able to read and write Japanese. Women were excluded if they had previous CS or serious medical conditions.	FMU	Hospital	Unplanned CS, instrumental vaginal delivery, augmentation of labor, episiotomy, APGAR score < 7 at one minute, APGAR score < 7 at five minutes, umbilical cord arterial pH, and breastfeeding initiation.	Part of the study
Prelec et al., 2014 [50]	Prospective case-control	Slovenia	497 women (154 for MW-led care and 343 for OB-led care)	Nulliparous women with singleton pregnancy at 37 to 41 + 3 weeks in cephalic presentation admitted in spontaneous active labor. Women were excluded if abnormal fetal heartbeat was detected.	AMU	Hospital	Unplanned CS, instrumental vaginal delivery, augmentation of labor, NO2 analgesia, narcotics use, no pain relief, 1st/2nd-degree perineal tear, 3rd/4th-degree perineal tear, episiotomy, PPH, blood transfusion, manual removal of placenta, physiological management of 3rd stage of labor, APGAR score < 7 at one minute, APGAR score < 7 at five minutes, NICU admission, need for resuscitation, need for ventilation, and breastfeeding.	-
Tracy et al., 2014 [51]	Cross-sectional	Australia	1379 women (482 for MW-led care and 897 for OB-led care)	Nulliparous 20- to 34-year-old women pregnant with singleton term normal weight fetus in cephalic-vertex presentation. Women with medical disorders, obstetric complications, or post-term pregnancy were excluded.	AMU	Hospital	Unplanned CS, instrumental vaginal delivery, augmentation of labor, epidural/spinal analgesia, episiotomy, APGAR score < 7 at five minutes, and NICU admission.	-
Hiraizumi et al., 2013 [52]	Retrospective cohort	Japan	508 women (291 for MW-led care and 217 for OB-led care)	Women with singleton term pregnancy in cephalic-vertex presentation presenting in labor or after rupture of membranes. Women were excluded if they had pre-existing or pregnancy-related DM or hypertension, complicated pregnancy (placenta previa, post-term pregnancy, polyhydramnios/oligohydramnios, IUGR, or fetal macrosomia), abnormal obstetric or gynecological history (previous CS, cephalopelvic disproportion, anal sphincter injury, manual removal of placenta, PPH > 1 L, blood transfusion, severe pre-eclampsia, uterine or adnexal anomaly, uterine myoma, or in vitro fertilization), medical disorder (e.g., renal disease, epilepsy, anemia, or idiopathic thrombocytopenia), or BMI > 28 (or >25 pre-pregnancy BMI).	Home or AMU	Hospital	Unplanned CS, instrumental vaginal delivery, augmentation of labor, 3rd/4th degree perineal tear, PPH, maternal infection/fever, and asphyxia.	-
Burns et al., 2012 [53]	Prospective cohort	The United Kingdom	8924 women (4794 for MW-led care and 4130 for OB-led care)	Women with an uncomplicated singleton term pregnancy in cephalic presentation admitted in labor and planning to use a birthing pool. Women were excluded in cases of pre-existing diseases or pregnancy complications.	Home, FMU, or AMU	Hospital	Unplanned CS, instrumental vaginal delivery, augmentation of labor, epidural/spinal analgesia, hydrotherapy pain relief, intact perineum, 1st/2nd-degree perineal tear, 3rd/4th-degree perineal tear, vaginal tear, labial tear, episiotomy, PPH, manual removal of placenta, physiological management of 3rd stage of labor, duration of labor, APGAR score < 7 at five minutes, NICU admission, need for resuscitation, need for ventilation, and shoulder dystocia.	-
Gaudineau et al., 2012 [54]	Case-control	France	1206 women (316 for MW-led care and 890 for OB-led care)	Women with a singleton term pregnancy in cephalic presentation. Women were excluded if they had pre-existing or gestational diabetes, complicated pregnancy (placenta praevia, IUGR, post-term pregnancy, induction of labor, PROM, or abnormal 30-minute cardiotocogram), medical disorder (severe disease or toxemia), or abnormal obstetric history (previous CS or cephalopelvic disproportion leading to difficult vaginal delivery).	AMU	Hospital	Unplanned CS, instrumental vaginal delivery, epidural/spinal analgesia, general anesthesia, no pain relief, intact perineum, 1st/2nd-degree perineal tear, 3rd/4th-degree perineal tear, episiotomy, PPH, five-minute APGAR score, umbilical cord arterial pH, and breastfeeding initiation.	-
Begley et al., 2011 and 2009 [55,56]	RCT	Ireland	1653 women (1101 for MW-led care and 552 for OB-led care)	Pregnant women from 16 to 40 years of age with BMI 18 to 29 before 24 weeks of gestation. Women were excluded if they had medical conditions (neurological, cardiovascular, respiratory, renal, gastrointestinal, endocrine, musculoskeletal, infective, hematological, or infective disease), psychological disorder, abnormal obstetric history (recurrent miscarriage, mid-trimester miscarriage, stillbirth, CS, preterm birth, uterine rupture, IUGR, moderate to severe pre-eclampsia, eclampsia, placental abruption, PPH, manual removal of the placenta, 3rd/4th degree tear, shoulder dystocia, hypoxic ischemic encephalopathy, neonatal death), abnormal gynecological history (uterine surgery, cone biopsy without subsequent vaginal term delivery, two Letz procedures uterine fibroids, uterine anomaly, cervical cerclage, perineal reconstruction, or infertility), parity > 5, latex allergy, illegal drugs use, smoking (≥20 cigarettes per day), or height < 152 cm.	AMU	Hospital	Unplanned CS, instrumental vaginal delivery, epidural/spinal analgesia, augmentation of labor, NO2 analgesia, general aneshesia, narcotics use, local analgesia/pudendal block, hydrotherapy pain relief, intact perineum, 1st/2nd-degree perineal tear, 3rd/4th degree perineal tear, episiotomy, PPH, manual removal of placenta, physiological management of 3rd stage of labor, maternal ICU admission, maternal infection/fever, duration of labor, umbilical cord arterial pH < 7.1, transfer to specialist neonatal care, NICU admission, meconium-stained fluid, need for resuscitation, need for ventilation, shoulder dystocia, breastfeeding initiation, and intrapartum or neonatal mortality.	Part of the study
Bernitz et al., 2011 and 2016 [57,58]	RCT	Norway	1111 women (412 for MW-led care and 699 for OB-led care)	Healthy women with a singleton pregnancy between 36 and 42 weeks of gestation in a cephalic presentation who presented in spontaneous labor. Women were excluded if they had medical conditions known to influence the pregnancy, previous complicated delivery or uterine surgery, pre-pregnancy BMI > 32 kg/m^2^, or smoking > 10 cigarettes per day.	AMU	Hospital	Unplanned CS, CS of suspected fetal distress, CS of non-progressive labor, instrumental vaginal delivery, epidural/spinal analgesia, augmentation of labor, NO2 analgesia, acupuncture pain relief, 3rd/4th degree perineal tear, episiotomy, PPH, APGAR score < 7 at five minutes, umbilical cord arterial pH < 7.1, and NICU admission.	-
Brocklehurst et al., 2011 [59]	Prospective cohort	The United Kingdom	64,538 women (44,832 for MW-led care and 19,706 for OB-led care)	Healthy women with uncomplicated singleton term pregnancy in cephalic presentation planning for vaginal delivery. Women were excluded if they had pre-existing or pregnancy-related hypertension or diabetes, pregnancy complications (recurrent antepartum hemorrhage, placenta previa, placental abruption, PROM, anemia, IUGR, IUFD, oligohydramnios/polyhydramnios, abnormal fetal heart rate, or induction of labor), abnormal obstetric or gynecological history (previous CS, hysterotomy, myomectomy, uterine rupture, placental abruption with adverse outcome, pre-eclampsia requiring preterm delivery, eclampsia, primary PPH requiring transfusion or additional treatment, manual removal of placenta, shoulder dystocia, unexplained stillbirth/neonatal death, or death of intrapartum complications), medical conditions (cardiovascular, respiratory, neurological, renal, gastrointestinal, endocrine, immunological, hematological, or infective), psychiatric disorder, alcohol/illicit drugs abuse, or no antenatal care.	Home, FMU, or AMU	Hospital	Unplanned CS, instrumental vaginal delivery, epidural/spinal analgesia, augmentation of labor, general anesthesia, hydrotherapy pain relief, 3rd/4th degree perineal tear, episiotomy, blood transfusion, physiological management of 3rd stage of labor, APGAR score < 7 at five minutes, transfer to specialist neonatal care, meconium stained fluid, breastfeeding initiation, and intrapartum or neonatal mortality.	-
Davis et al., 2011 and 2012 [60,61]	Retrospective cohort	New Zealand	16,147 women (4699 for MW-led care and 11,448 for OB-led care)	Women with singleton term pregnancy in cephalic presentation planned for vaginal delivery. Women were excluded if they had pre-existing or pregnancy-related DM or hypertension, pregnancy complication (post-term pregnancy, induction of labor, antepartum stillbirth, or antenatal transfer of care), abnormal obstetric history (previous CS, PPH > 1 L, Rhesus or ABO incompatibility, or stillbirth), medical disorder (neurological, cardiac, respiratory, renal, musculoskeletal, or thyroid disease), or illegal drugs or alcohol abuse.	Home or FMU	Hospital	Unplanned CS, instrumental vaginal delivery, PPH, physiological management of 3rd stage of labor, duration of labor, and intrapartum or neonatal mortality.	-
Gottvall et al., 2011 [62]	Retrospective cohort	Sweden	11,937 women (2555 for MW-led care and 9382 for OB-led care)	Women with singleton pregnancy. Women were excluded if they had a BMI > 29, previous CS, previous perinatal death, old primigravida (>40 years), DM, hypertension, epilepsy, or smoking.	AMU	Hospital	Unplanned CS, instrumental vaginal delivery, epidural/spinal analgesia, NO2 analgesia, narcotics use, acupuncture pain relief, no pain relief, episiotomy, PPH, APGAR score < 7 at five minutes, meconium stained fluid, asphyxia, and intrapartum or neonatal mortality.	Part of the study
Overgaard et al., 2011 [63]	Prospective cohort	Denmark	1678 women (839 for MW-led care and 839 for OB-led care)	Healthy women with uncomplicated singleton term pregnancy in cephalic presentation planning for vaginal delivery. Women were excluded if they had pre-existing or pregnancy-related hypertension or diabetes, pregnancy complications (recurrent antepartum hemorrhage, placenta previa, placental abruption, PROM, anemia, IUGR, IUFD, oligohydramnios/polyhydramnios, abnormal fetal heart rate, or induction of labor), Abnormal obstetric or gynecological history (previous CS, hysterotomy, myomectomy, uterine rupture, placental abruption with adverse outcome, pre-eclampsia requiring preterm delivery, eclampsia, primary PPH requiring transfusion or additional treatment, manual removal of placenta, shoulder dystocia, unexplained stillbirth/neonatal death, or death of intrapartum complications), medical conditions (cardiovascular, respiratory, neurological, renal, gastrointestinal, endocrine, immunological, hematological, or infective), psychiatric disorder, alcohol/illicit drugs abuse, or no antenatal care.	FMU	Hospital	Unplanned CS, instrumental vaginal delivery, epidural/spinal analgesia, augmentation of labor, hydrotherapy pain relief, intact perineum, 1st/2nd-degree perineal tear, 3rd/4th degree perineal tear, PPH, severe maternal morbidity, APGAR score < 7 at one minute, APGAR score < 7 at five minutes, NICU admission, meconium stained fluid, asphyxia, shoulder dystocia, and intrapartum or neonatal mortality.	-
Browne et al., 2010 [64]	Retrospective cohort	The United States	2819 women (1082 for MW-led care and 1737 for OB-led care)	Nulliparous women having a term pregnancy in cephalic presentation ended with spontaneous vaginal delivery. Women were excluded if they had CS, assisted vaginal delivery, stillbirth, neonatal death, or labial lacerations only.	AMU	Hospital	Epidural/spinal analgesia.	-
Eide et al., 2009 [65]	Prospective cohort	Norway	453 women (252 for MW-led care and 201 for OB-led care)	Healthy nulliparous women with uncomplicated pregnancies on regular antenatal care who were admitted in labor beyond 36 weeks of gestation. Women were excluded if they had post-term pregnancy, fetal anomaly, fetal/placental disease, PROM, hemophilia, thrombophilia, desire for epidural analgesia before admission to the labor ward, or illegal drugs or alcohol abuse.	AMU	Hospital	Unplanned CS, instrumental vaginal delivery, epidural/spinal analgesia, NO2 analgesia, narcotics use, acupuncture pain relief, local analgesia/pudendal block, hydrotherapy pain relief, 3rd/4th degree perineal tear, and episiotomy.	-
Suzuki et al., 2009 [66]	Retrospective cohort	Japan	548 women (459 for MW-led care and 89 for OB-led care)	Healthy women With uncomplicated pregnancy. Women were excluded if they had pre-existing or pregnancy-related DM or hypertension, complicated pregnancy (polyhydramnios/oligohydramnios, placenta previa, IUGR, fetal macrosomia, or pre-eclampsia), medical disorder (e.g., idiopathic thrombocytopenia, renal disease, epilepsy, anemia), abnormal obstetric or gynecological history (previous CS, cephaloplvic disproportion, anal sphincter injury, manual removal of placenta, PPH > 1 L with blood transfusion, severe pre-eclampsia, uterine or adnexal anomaly, uterine myoma, or in vitro fertilization), or BMI > 28 (or pre-pregnancy BMI > 25).	AMU	Hospital	Unplanned CS, instrumental vaginal delivery, PPH, APGAR score < 7 at one minute, APGAR score < 7 at five minutes, one minute APGAR score, five-minute APGAR score, umbilical cord arterial PH < 7.1, and umbilical cord arterial PH.	-
Maassen et al., 2008 [67]	Retrospective cohort	The Netherlands	107,667 women (87,817 for MW-led care and 19,850 for OB-led care)	Healthy women with uncomplicated pregnancy. Women were excluded if they had complicated pregnancy (hospital admission, polyhydramnios, antepartum hemorrhage, placenta previa, placental abruption, IUGR, pre-eclampsia, hyperemesis gravidarum, tocolysis, blood group incompatibility, congenital viral infection, or PAP smear > IIIa), pre-existing or pregnancy-related hypertension or DM, medical conditions (cardiovascular, neurological, renal, gastrointestinal, endocrine, dermatological, orthopedic, or other systemic disorder, or HIV infection), neurosurgery or orthopedic surgery, psychiatric disorder, abnormal obstetric or gynecological history (narrow pelvic outlet, previous CS, anal sphincter injury, manual removal of placenta, PPH > 1 L needing a blood transfusion, severe pre-eclampsia, molar pregnancy/choriocarcinoma, insulin-controlled gestational diabetes, uterine or adnexal anomaly, uterine myoma, endometriosis, cervical or pelvic floor surgery), or illegal drugs use.	Home or AMU	Hospital	Unplanned CS and instrumental vaginal delivery.	-
Ryan et al., 2005 [68]	Retrospective cohort	Australia	3683 women (720 for MW-led care and 2963 for OB-led care)	Women with singleton term pregnancy in cephalic presentation planning for vaginal delivery. Women were excluded if they had IUGR, had a major medical condition, were using insulin, were using two oral medications for pre-eclampsia, or were illegal drug users.	AMU	Hospital	Unplanned CS, instrumental vaginal delivery, epidural/spinal analgesia, augmentation of labor, NO2 analgesia, narcotics use, intact perineum, 1st/2nd-degree perineal tear, episiotomy, PPH, manual removal of placenta, physiological management of 3rd stage of labor, duration of labor, APGAR score < 7 at one minute, APGAR score < 7 at five minutes, NICU admission, need for resuscitation, need for ventilation, breastfeeding initiation, and intrapartum or neonatal mortality.	-
Rana et al. 2003 [69]	Retrospective cohort	Nepal	988 women (550 for MW-led care and 438 for OB-led care)	Women with uncomplicated pregnancies who received complete antenatal care and presented in labor.	AMU	Hospital	Unplanned CS, instrumental vaginal delivery, augmentation of labor, intact perineum, 3rd/4th degree perineal tear, episiotomy, five-minute APGAR score. NICU admission, meconium-stained fluid, and intrapartum or neonatal mortality.	-

Abbreviations: AMU: Alongside midwifery unit; BMI: Body mass index; CS: Cesarian section; DM: Diabetes mellitus; FMU: Freestanding midwifery unit; Hb: Hemoglobin; HIV: Human immunodeficiency virus; ICU: Intensive care unit; IUFD: Intrauterine fetal death; IUGR: Intrauterine growth restriction; MW: Midwife; NICU: Neonatal Intensive care unit; OB: Obstetrician; PPH: Postpartum hemorrhage; PROM: Premature rupture of membranes; RCT: Randomized controlled trial.

**Table 2:** Baseline characteristics of the enrolled participants.

Study ID	Age (Years)		Nulliparous		Gestational Age (Weeks)		Amniotomy		Induction of Labor		BMI (kg/m^2^)		Race						Married\Partnered		University or Higher Education		Working		Habits						Male Newborn		Newborn Birth Weight (kg)	
													Caucasian		African		Asian								Smoking		Alcohol		Illicit Drugs					
	Mean ± SD		*n* (%)		Mean ± SD		*n* (%)		*n* (%)		Mean ± SD		*n* (%)		*n* (%)		*n* (%)		*n* (%)		*n* (%)		*n* (%)		*n* (%)		*n* (%)		*n* (%)		*n* (%)		Mean ± SD	
	MW-Led	OB-Led	MW-Led	OB-Led	MW-Led	OB-Led	MW-Led	OB-Led	MW-Led	OB-Led	MW-Led	OB-Led	MW-Led	OB-Led	MW-Led	OB-Led	MW-Led	OB-Led	MW-Led	OB-Led	MW-Led	OB-Led	MW-Led	OB-Led	MW-Led	OB-Led	MW-Led	OB-Led	MW-Led	OB-Led	MW-Led	OB-Led	MW-Led	OB-Led
Sorbara et al., 2024 [22]	<19: 272 (1.2) * 20–24: 1921 (8.3) 25–29: 7715 (33.4) 30–34: 9801 (42.4) 35–39: 3039 (13.1) ≥40: 376 (1.6)	<19: 2121 (2.6) * 20–2: 8802 (10.8) 25–29: 24,933 (30.5) 30–34: 30,909 (37.8) 35–39: 12,161 (14.9) ≥40: 2945 (3.6)	23,124 (100)	81,871 (100)	40 ± 1.5	39 ± 1.5	-	-	-	-	<18.5: 918 (4) * 18.5–24.9: 13,771 (59.6) 25–29.9: 5301 (22.9) ≥30: 3134 (13.6)	<18.5: 4948 (6) * 18.5–24.9: 46,457 (56.7) 25–29.9: 17,842 (21.8) ≥30: 12,624 (15.4)	-	-	-	-	-	-	-	-	-	-	-	-	856 (3.7)	5398 (6.6)	565 (2.4)	1819 (2.2)	223 (1)	1151 (1.4)	-	-	-	-
Palau-Costafreda et al., 2023 [23]	33 ± 3.7	30 ± 4.5	149 (58.4)	262 (42.1)	37–39 + 6: 105 (41.3) * 40–40 + 6: 97 (38.2) 41–41 + 6: 48 (18.9) ≥42: 4 (1.6)	37–39 + 6: 280 (53.9) * 40–40 + 6: 141 (27.2) 41–41 + 6: 96 (18.5) ≥42: 2 (0.4)	-	-	-	-	<18.5: 10 (3.9) * 18.5–24.9: 208 (81.6) 25–29.9: 29 (11.4) ≥30: 8 (3.2)	<18.5: 23 (3.7) * 18.5–24.9: 376 (60.4) 25–29.9: 147 (23.6) ≥30: 77 (12.4)	218 (85.5)	314 (50.4)	18 (7.1)	236 (37.9)	3 (1.2)	19 (3)	-	-	-	-	-	-	-	-	-	-	-	-	-	-	-	-
Stoll et al., 2023 [24]	-	-	-	-	-	-	-	-	2986 (6.4)	10,758 (14)	-	-	-	-	-	-	-	-	-	-	-	-	-	-	-	-	-	-	-	-	-	-	<2.5: 448 (1) *	<2.5: 2096 (2.7) *
Martin-Arribas et al., 2022 [25]	31.3 ± 5.1	31.8 ± 4.9	5164 (47.6)	401 (57.9)	39.5 ± 1.1	39.6 ± 1.2	-	-	2745 (25.6)	329 (55.5)	-	-	8043 (74.3)	535 (77.2)	1379 (12.7)	71 (10.2)	18 (2.6)	293 (2.5)	-	-	2970 (27.4)	217 (31.3)	-	-	-	-	-	-	-	-	-	-	-	-
Poskienc et al., 2021 [26]	28.9 ± 0.5	28.6 ± 4.1	44 (23.9)	610 (36.7)	39.2 ± 1.1	39.4 ± 1.0	39 (21.2)	237 (14.2)	69 (37.5)	827 (49.6)	27.0 ± 3.5	27.8 ± 4.1	-	-	-	-	-	-	-	-	-	-	-	-	-	-	-	-	-	-	-	-	3.48 ± 0.35	3.54 ± 0.30
Tietjen et al., 2021 [27]	-	-	219 (56)	216 (55.2)	-	-	-	-	-	-	-	-	-	-	-	-	-	-	-	-	-	-	-	-	-	-	-	-	-	-	-	-	3.48 ± 0.39	3.46 ± 0.40
Merz et al., 2020 [28]	32.9 ± 4.4	32.1 ± 5.1	281 (45.9)	281 (45.9)	-	-	-	-	-	-	≥25: 421 (68.8)	≥ 25: 196 (32)	-	-	-	-	-	-	-	-	-	-	-	-	-	-	-	-	-	-	-	-	3.56 ± 0.43	3.47 ± 0.42
Welffens et al., 2019 [29]	31.9 ± 0.2	29.8 ± 0.3	367 (62.2)	176 (44.7)	-	-	-	-	96 (16.3)	120 (30.5)	<18.5: 33 (5.6) *˜ 18.5–24.9: 430 (72.9) 25–29.9: 72 (12.2) ≥30: 55 (9.3)	<18.5: 7 (1.8) *˜ 18.5–24.9: 221 (56.1) 25–29.9: 108 (27.4) ≥30: 58 (14.7)	408 (89.3)	167 (43.1)	6 (1.3)	63 (16.3)	-	-	-	-	-	-	-	-	-	-	-	-	0 (0)	0 (0)	-	-	-	-
Wiegerinck et al., 2020 [30]	30.5 ± 4.7	30.9 ± 5.4	88,610 (42.9)	24,056 (45.8)	39.6 ± 1.0‘‘‘	39.3 ± 1.1‘‘‘	-	-	0 (0)	0 (0)	-	-	-	-	-	-	-	-	-	-	-	-	-	-	-	-	-	-	-	-	104,158 (50.4)	26,778 (50.9)	3.55 ± 0.44	3.44 ± 0.48
Isaline et al., 2019 [31]	32.3 ± 0.6	29.6 ± 0.9	42 (71.2)	14 (46.7)	39.3 ± 0.3	39.1 ± 0.5	23 (39)	16 (53.3)	19 (32.2)	7 (23.3)	22.2 ± 0.4	24.1 ± 0.7	-	-	-	-	-	-	-	-	44 (74.6)	11 (36.7)	-	-	-	-	-	-	-	-	-	-	3.31 ± 0.07	3.18 ± 0.13
Koto et al., 2019 [32]	-	-	276 (36.7)	4464 (38.9)	-	-	-	-	-	-	<18.5: 21 (2.8) * 18.5–24.9: 440 (58.4) 25–29.9: 139 (18.5) ≥30: 153 (20.3)	<18.5: 441 (3.8) 18.5–24.9: 4668 (40.7) 25–29.9: 2350 (20.5) ≥30: 4016 (35)	573 (77.6)	6053 (53.1)	-	-	-	-	485 (64.4)	5516 (48.1)	101 (13.5)	410 (3.6)	-	-	58 (7.7)	1748 (15.2)	-	-	-	-	-	-	>2.5: 753 (100) *	>2.5: 11,475 (100) *
Pérez-Martínez et al., 2019 [33]	31.0 ± 5.2	30.9 ± 5.5	495 (47.6)	546 (52.4)	-	-	-	-	-	-	-	-	-	-	-	-	-	-	-	-	-	-	-	-	-	-	-	-	-	-	637 (48.7)	651 (49.6)	-	-
Souter et al., 2019 [34]	29.8 ± 5.1	29.5 ± 5.1	1710 (44.8)	9096 (47.2)	≥41: 778 (20.4) *	≥41: 2826 (14.7) *	1432 (37.5)	9170 (47.6)	524 (13.7)	4032 (20.9)	Obese: 60 (1.6) *	Obese: 60 (0.3) *	2797 (73.3)	12,028 (62.4)	171 (4.5)	588 (3)	449 (11.8)	4467 (23.2)	-	-	-	-	-	-	0 (0)	0 (0)	0 (0)	0 (0)	0 (0)	0 (0)	-	-	3.52 ± 0.43	3.45 ± 0.43
Bartuseviciene et al., 2018 [35]	28 ± 4.7	29.4 ± 4.9	455 (50)	916 (52.1)	39.5 ± 0.9	39.5 ± 0.9	236 (25.9)	613 (34.9)	-	-	-	-	-	-	-	-	-	-	-	-	-	-	-	-	-	-	-	-	-	-	-	-	3.51 ± 0.39	3.54 ± 0.42
Carlson et al., 2018 [36]	18–29: 527 (89.1) * 30–40: 63 (10.7)	18–29: 518 (69.2) * 30–40: 231 (30.8)	590 (100)	749 (100)	37–37 + 6: 34 (5.8) * 38–38 + 6: 110 (18.6) 39–39 + 6: 164 (27.8) 40–40 + 6: 205 (34.7) 41–41 + 6: 77 (13.1)	37–37 + 6: 60 (8) * 38–38 + 6: 127 (17) 39–39 + 6: 263 (35.1) 40–40 + 6: 235 (31.4) 41–41 + 6: 64 (8.5)	285 (48.3)	389 (51.9)	-	-	<25: 91 (15.4) * 25–29.9: 242 (41) ≥30: 257 (45.6)	<25: 127 (17) * 25–29.9: 333 (44.5) ≥30: 289 (38.6)	422 (71.6)	587 (78.4)	116 (19.7)	77 (10.3)	-	-	271 (45.9)	507 (67.7)	-	-	-	-	68 (11.5)	64 (8.5)	1 (0.2)	1 (0.1)	4 (0.7)	5 (0.6)	-	-	<2.5: 12 (2) * 2.5–3.99: 563 (95.4) ≥4: 14 (2.4)	<2.5: 23 (3.1) * 2.5–3.99: 702 (93.7) ≥4: 22 (2.9)
Hua et al., 2018 [37]	28.7 ± 3.2	28.5 ± 3.7	451 (100)	1117 (100)	-	-	-	-	-	-	-	-	-	-	-	-	-	-	-	-	341 (75.6)	832 (74.5)	370 (82)	907 (81.2)	-	-	-	-	-	-	-	-	-	-
Wiegerinck et al., 2018 [38]	30.4 ± 5.0	31.4 ± 5.5	21,074 (45.1)	4376 (41.2)	40 ± 0.7	40 ± 0.7	-	-	0 (0)	0 (0)	-	-	-	-	-	-	-	-	-	-	-	-	-	-	-	-	-	-	-	-	23,668 (50.6)	5453 (51.3)	3.55 ± 0.46	3.47 ± 0.50
Altman et al., 2017 [39]	28.1	29	151 (41.8)	530 (49.1)	-	-	170 (47.09)	564 (52.22)	83 (23)	501 (46.4)	27.7	29.4	250 (69.3)	815 (75.5)	12 (3.3)	40 (3.7)	17 (4.7)	67 (6.2)	208 (57.6)	681 (63.1)	-	-	-	-	-	-	27 (7.5)	87 (8.1)	26 (7.2)	50 (4.6)	-	-	-	-
Bodner-Adler et al., 2017 and 2004 [40,41]	28 ± 5.9	28 ± 5.9	578 (27)	515 (26)	40 ± 1.5	40 ± 1.5	237 (11)	269 (14)	-	-	-	-	-	-	-	-	-	-	-	-	-	-	-	-	-	-	-	-	-	-	-	-	3.40 ± 0.45	3.42 ± 0.45
Schroeder et al., 2017 [42]	-	-	-	-	-	-	0.13 ± 0.3 §	0.30 ± 0.4 §	-	-	-	-	93 (43.3)	20 (11.4)	10 (4.7)	5 (2.9)	37 (17.2)	127 (73)	131 (92.9)	97 (93.2)	-	-	-	-	-	-	-	-	-	-	-	-	-	-
Voon et al., 2017 [43]	28.9 ± 4.7	28.8 ± 5.1	67 (39.4)	63 (31.8)	-	-	-	-	35 (20.6)	50 (25.3)	-	-	-	-	-	-	170 (100)	198 (100)	-	-	-	-	95 (55.9)	82 (41.4)	9 (5.3)	19 (9.6)	-	-	-	-	-	-	<2.5: 5 (2.9) *	<2.5: 9 (4.5) *
Thiessen et al., 2016 [44]	≤19: 225 (5.7) * 20–34: 3214 (81) ≥35: 531 (13.4)	≤19: 6279 (8.7) * 20–34: 55,356 (76.9) ≥35: 10,374 (14.4)	1109 (27.9)	3123 (40.1)	39.7 ± 1.1	39.4 ± 1.2	-	-	-	-	-	-	-	-	-	-	-	-	-	-	-	-	-	-	-	-	-	-	-	-	-	-	3.6 ± 0.5	3.5 ± 0.5
Thornton et al., 2016 [45]	29 ± 5.2	26.8 ± 5.9	4078 (46.5)	1006 (39.8)	39.9 ± 1.1	39.7 ± 1.1	-	-	0 (0)	0 (0)	-	-	7490 (85.4)	1871 (74)	428 (4.9)	275 (10.9)	220 (2.5)	131 (5.2)	7907 (90.1)	1888 (74.7)	-	-	-	-	-	-	-	-	0 (0)	0 (0)	-	-	3.55 ± 0.43	3.45 ± 0.44
De Jonge et al., 2015 [46]	< 25: 21,695 (12.7) * 25–34: 117,717 (69.1) >35: 31,011 (18.2)	< 25: 6367 (11.9) * 25–34: 33,018 (62) >35: 13,910 (26.1)	76,435 (44.8)	24,251 (45.5)	37–37 + 6: 7011 (4.1) * 38–40 + 6: 125,359 (73.6) 41–41 + 6: 38,069 (22.3)	37–37 + 6: 4023 (7.5) * 38–40 + 6: 38,391 (72) 41–41 + 6: 10,886 (20.4)	-	-	0 (0)	0 (0)	-	-	-	-	-	-	-	-	-	-	-	-	-	-	-	-	-	-	-	-	-	-	-	-
Walters et al., 2015 [47]	31.2 ± 3.9	29.7 ± 4.9	-	-	-	-	-	-	-	-	-	-	-	-	-	-	-	-	-	-	-	-	-	-	-	-	-	-	-	-	-	-	-	-
Homer et al., 2014 [48]	30.1 ± 5.3	29.2 ± 5.7	9458 (62.2)	149,459 (61.5)	39.8 ± 1.1	39.5 ± 1.1	-	-	0 (0)	0 (0)	-	-	-	-	-	-	-	-	-	-	-	-	-	-	-	-	-	-	-	-	-	-	-	-
Iida et al., 2014 [49]	33.2 ± 4.3	31.7 ± 4.3	60 (40.3)	68 (51.9)	-	-	9 (6)	33 (25.2)	0 (0)	14 (10.7)	-	-	-	-	-	-	-	-	-	-	-	-	-	-	-	-	-	-	-	-	-	-	-	-
Prelec et al., 2014 [50]	28.5	28.7	154 (100)	343 (100)	-	-	85 (55.2)	175 (51)	0 (0)	0 (0)	-	-	-	-	-	-	-	-	150 (97.4)	331 (96.5)	84 (54.5)	161 (53.2)	-	-	-	-	-	-	-	-	82 (53.2)	176 (51.3)	3.36	3.42
Tracy et al., 2014 [51]	-	-	-	-	-	-	-	-	265 (55)	620 (69)	-	-	-	-	-	-	-	-	-	-	-	-	-	-	-	-	-	-	-	-	-	-	-	-
Hiraizumi et al., 2013 [52]	32.6 ± 4.5	32.9 ± 4.8	71 (24)	56 (26)	-	-	-	-	0 (0)	0 (0)	-	-	-	-	-	-	-	-	-	-	-	-	-	-	-	-	-	-	-	-	-	-	-	-
Burns et al., 2012 [53]	29.4 ± 5.6	29.4 ± 5.6	2520 (52.6)	2433 (59)	39.8 ± 1.1	39.7 ± 1.1	669 (14)	963 (23.3)	56 (1.2)	170 (4.1)	-	-	-	-	-	-	-	-	-	-	-	-	-	-	-	-	-	-	-	-	-	-	3.53 ± 0.44	3.54 ± 0.43
Gaudineau et al., 2012 [54]	29 ± 5	28.7 ± 5.4	142 (44.9)	361 (40.5)	39.5 ± 1.1	39.2 ± 1.2	-	-	0 (0)	0 (0)	-	-	-	-	-	-	-	-	-	-	-	-	-	-	-	-	-	-	-	-	-	-	3.32 ± 0.39	3.35 ± 0.43
Begley et al., 2011 and 2009 [55,56]	29 ± 4.9	28.7 ± 5	565 (51.3)	276 (50)	-	-	135 (54.4)	65 (47.1)	254 (23.1)	132 (23.9)	23.9 ± 0.2	23.9 ± 0.1	-	-	-	-	-	-	664 (60.3)	312 (56.5)	-	-	-	-	-	-	-	-	0 (0)	0 (0)	-	-	3.54 ± 0.52	3.5 ± 0.53
Bernitz et al., 2011 and 2016 [57,58]	<25: 103 (25) * 25–35: 263 (63.8) >35: 46 (11.2)	<25: 164 (19.8) * 25–35: 451 (54.4) >35: 84 (10.1)	278 (67.5)	469 (67.1)	-	-	-	-	0 (0)	0 (0)	-	-	-	-	-	-	-	-	391 (94.9)	666 (95.3)	202 (49)	357 (51.1)	-	-	-	-	-	-	-	-	-	-	-	-
Brocklehurst et al., 2011 [59]	29.5 ± 5.7	28.2 ± 6	18,105 (40.7)	10,626 (54)	39.8 ± 1.0	39.8 ± 1.1	-	-	-	-	24 ± 3.7	24 ± 4.0	39,751 (88.8)	16,068 (81.7)	726 (1.6)	670 (3.4)	-	-	41,514 (93.6)	17,097 (88.2)	-	-	-	-	-	-	0 (0)	0 (0)	0 (0)	0 (0)	-	-	-	-
Davis et al., 2011 and 2012 [60,61]	28.9 ± 5.9	28.3 ± 6	1534 (32.6)	5544 (48.2)	-	-	-	-	0 (0)	0 (0)	-	-	3263 (69.3)	7476 (65)	-	-	131 (2.8)	775 (6.7)	-	-	-	-	-	-	-	-	0 (0)	0 (0)	0 (0)	0 (0)	-	-	-	-
Gottvall et al., 2011 [62]	32.1	31.2	1264 (49.5)	4878 (52)	<37: 50 (2) * 37–41: 2435 (95.3) ≥42: 68 (2.7)	<37: 385 (4.1) * 37–41: 8705 (92.8) ≥42: 284 (3)	-	-	326 (12.8)	1563 (16.7)	-	-	-	-	-	-	-	-	1566 (61.3)	5770 (61.5)	1914 (74.9)	5057 (53.9)	-	-	0 (0)	0 (0)	-	-	-	-	-	-	<2.5: 40 (1.6) 2.5–4.5: 2417 (94.6) >4.5: 96 (3.8)	<2.5: 306 (3.3) 2.5–4.5: 8769 (93.5) >4.5: 295 (3.1)
Overgaard et al., 2011 [63]	16–20: 24 (2.9) * 21–35: 731 (87.1) >35: 84 (10)	16–20: 25 (3) * 21–35: 716 (85.3) >35: 98 (11.7)	215 (25.6)	215 (25.6)	-	-	-	-	0 (0)	0 (0)	<18.5: 17 (2.1) * 18.5–24.9: 528 (62.9) 25–29.9: 226 (26.9) ≥30: 68 (8.1)	<18.5: 22 (2.6) * 18.5–24.9: 530 (63.2) 25–29.9: 219 (26.1) ≥30: 68 (8.1)	-	-	-	-	-	-	815 (97.1)	819 (97.6)	368 (43.9)	367 (43.7)	679 (80.9)	708 (84.4)	155 (18.5)	155 (18.5)	-	-	-	-	-	-	3.64	3.64
Browne et al., 2010 [64]	24.4 ± 5.7	31.5 ± 5.4	1082 (100)	1737 (100)	-	-	-	-	-	-	-	-	250 (23.1)	1396 (80.4)	20 (1.9)	31 (1.8)	44 (4.1)	135 (7.8)	-	-	-	-	-	-	-	-	-	-	-	-	-	-	3.36 ± 0.44	3.42 ± 0.45
Eide et al., 2009 [65]	<29: 10 (4) * 20–24: 62 (24.6) 25–29: 109 (43.3) 30–34: 57 (22.6) >34: 14 (5.6)	<29: 12 (6) * 20–24: 56 (27.9) 25–29: 77 (38.3) 30–34: 44 (21.9) >34: 12 (6)	252 (100)	201 (100)	-	-	-	-	-	-	-	-	-	-	-	-	-	-	232 (92.1)	168 (83.6)	134 (53.2)	85 (42.3)	232 (92.1)	154 (76.6)	43 (17.1)	56 (27.9)	0 (0)	0 (0)	0 (0)	0 (0)	-	-	-	-
Suzuki et al., 2009 [66]	32 ± 5	33 ± 5	218 (47)	49 (55)	39.6 ± 1.0	39.6 ± 1.0	-	-	-	-	-	-	-	-	-	-	-	-	-	-	-	-	-	-	-	-	-	-	-	-	-	-	3.03 ± 0.42	3.06 ± 0.44
Maassen et al., 2008 [67]	-	-	33,187 (80.4)	8071 (19.6)	-	-	-	-	-	-	-	-	-	-	-	-	-	-	-	-	-	-	-	-	-	-	-	-	0 (0)	0 (0)	-	-	-	-
Ryan et al., 2005 [68]	<20: 2 (0.3) * 20–24: 62 (8.6) 25–29: 164 (22.8) 30–34: 302 (41.9) 35–39: 168 (23.3) >40: 22 (3.1)	<20: 91 (3.1) * 20–24: 498 (16.8) 25–29: 883 (29.8) 30–34: 955 (32.2) 35–39: 436 (14.7) >40: 100 (3.4)	366 (50.8)	1421 (48)	39–41: 594 (82.5) *	39–41: 2164 (73) *	-	-	56 (7.7)	613 (20.7)	-	-	-	-	-	-	-	-	651 (90.4)	2523 (85.2)	488 (67.8)	1284 (43.4)	-	-	53 (7.4)	355 (12)	-	-	0 (0)	0 (0)	-	-	≤3rd: 8 (1.1) ¶ > 3rd–≤25th: 115 (16) > 25th–≤75th: 381 (52.9) > 75th–≤ 97th: 190 (26,4) > 97th: 26 (3.6)	≤3rd: 86 (2.9) ¶ > 3rd–≤25th: 679 (23) > 25th–≤75th: 1499 (50.7) > 75th–≤ 97th: 610 (20.5) > 97th: 86 (2.9)
Rana et al., 2003 [69]	23‘‘‘	24‘‘‘	-	-	-	-	293 (53.2)	185 (42.3)	1 (0.2)	43 (9.9)	-	-	-	-	-	-	-	-	-	-	-	-	-	-	-	-	-	-	-	-	-	-	3.00	3.01

Abbreviations: BMI: Body mass index; MW: Midwife; OB: Obstetrician; SD: Standard deviation. * *n* (%), § mean ± SD, ‘‘‘ median, ¶ percentiles, ˜ pre-pregnancy value.

**Table 3:** Secondary outcomes synthesis results.

Outcome	No. of Studies	No. of Participants	Effect Estimate	95% CI	*p*-Value	Heterogeneity
Birth interventions						
CS of suspected fetal distress	3	59,846	RR = 0.49	[0.17, 1.41]	0.19	*p* < 0.001, I^2^ = 93%
CS of non-progressive labor	3	59,846	RR = 0.65	[0.30, 1.43]	0.29	*p* < 0.001, I^2^ = 94%
Augmentation of labor	19	599,504	RR = 0.61	[0.52, 0.71]	<0.001 *	*p* < 0.001, I^2^ = 99%
NO2 anesthesia	8	21,541	RR = 0.88	[0.73, 1.06]	0.18	*p* < 0.001, I^2^ = 95%
General anesthesia	6	96,733	RR = 0.60	[0.38, 0.96]	0.03 *	*p* < 0.001, I^2^ = 77%
Pudendal nerve block	3	4718	RR = 0.55	[0.29, 1.06]	0.07	*p* = 0.10, I^2^ = 57%
Narcotics use	6	18,582	RR = 0.84	[0.51, 1.38]	0.50	*p* < 0.001, I^2^ = 94%
Acupuncture pain relief	3	13,501	RR = 2.51	[1.45, 4.35]	0.001 *	*p* < 0.001, I^2^ = 97%
Hydrotherapy pain relief	6	80,863	RR = 1.85	[0.87, 3.96]	0.11	*p* < 0.001, I^2^ = 100%
No pain relief	7	22,575	RR = 1.59	[1.09, 2.32]	0.02 *	*p* < 0.001, I^2^ = 97%
Episiotomy	28	584,496	RR = 0.66	[0.59, 0.73]	<0.001 *	*p* < 0.001, I^2^ = 95%
Physiological management of 3rd stage	6	95,033	RR = 3.36	[2.03, 5.57]	<0.001 *	*p* < 0.001, I^2^ = 100%
Maternal outcomes						
Intact perineum	13	25,338	RR = 1.06	[0.98, 1.15]	0.16	*p* < 0.001, I^2^ = 80%
1st or 2nd-degree perineal tear	17	98,610	RR = 1.14	[0.69, 1.87]	0.61	*p* < 0.001, I^2^ = 100%
3rd or 4th-degree perineal tear	27	629,053	RR = 0.87	[0.74, 1.02]	0.08	*p* < 0.001, I^2^ = 89%
Vaginal tear	2	13,011	RR = 1.16	[1.03, 1.31]	0.01 *	*p* = 0.01, I^2^ = 85%
Labial tear	3	14,859	RR = 1.04	[0.93, 1.16]	0.53	*p* = 0.64, I^2^ = 0%
Manual removal of the placenta	9	297,395	RR = 0.54	[0.43, 0.69]	<0.001 *	*p* < 0.001, I^2^ = 79%
Blood transfusion	5	212,408	RR = 0.65	[0.43, 0.97]	0.03 *	*p* < 0.001, I^2^ = 80%
Maternal infection or fever	6	21,257	RR = 0.76	[0.62, 0.94]	0.01 *	*p* = 0.67, I^2^ = 0%
Severe maternal morbidity	3	248,517	RR = 0.72	[0.33, 1.59]	0.42	*p* = 0.003, I^2^ = 83%
Maternal ICU admission	3	348,709	RR = 0.35	[0.26, 0.48]	<0.001 *	*p* = 0.78, I^2^ = 0%
Duration of labor (hours)	7	33,980	MD =−0.07	[−0.77, 0.63]	0.84	*p* < 0.001, I^2^ = 97%
Duration of hospital stay (days)	5	4382	MD =−0.31	[−0.57, −0.06]	0.02 *	*p* < 0.001, I^2^ = 95%
Neonatal outcomes						
1-minute APGAR score	3	3175	MD = 0.10	[0.03, 0.18]	0.009 *	*p* = 0.23, I^2^ = 31%
5-minute APGAR score	6	83,028	MD = 0.03	[−0.01, 0.08]	0.13	*p* = 0.005, I^2^ = 70%
Umbilical cord arterial pH < 7.1	6	9382	RR = 0.77	[0.59, 0.98]	0.04 *	*p* = 0.77, I^2^ = 0%
Umbilical cord arterial pH	4	2730	MD = 0.00	[−0.01, 0.02]	0.87	*p* = 0.003, I^2^ = 78%
Meconium-stained amniotic fluid	6	91,767	RR = 0.68	[0.30, 1.53]	0.35	*p* < 0.001, I^2^ = 99%
Asphyxia	3	14,123	RR = 0.66	[0.45, 0.96]	0.03 *	*p* = 0.64, I^2^ = 0%
Need for resuscitation	6	113,827	RR = 0.80	[0.54, 1.19]	0.27	*p* < 0.001, I^2^ = 97%
Need for ventilation	6	27,399	RR = 0.97	[0.66, 1.43]	0.89	*p* < 0.001, I^2^ = 82%
Shoulder dystocia	5	36,685	RR = 0.79	[0.45, 1.36]	0.39	*p* = 0.002, I^2^ = 76%
Transfer to specialist neonatal care	5	68,807	RR = 0.72	[0.53, 0.97]	0.03 *	*p* = 0.001, I^2^ = 78%
NICU admission	17	175,224	RR = 0.75	[0.59, 0.94]	0.01 *	*p* < 0.001, I^2^ = 78%
Breastfeeding initiation	15	300,274	RR = 1.14	[1.06, 1.23]	<0.001 *	*p* < 0.001, I^2^ = 99%

Abbreviations: CI: Confidence interval; CS: Cesarian section; ICU: Intensive care unit; MD: Mean difference; NICU: Neonatal intensive care unit; RR: Risk ratio. * Significant *p*-value.

## Data Availability

Data are available from the corresponding author upon reasonable request.

## Supplementary Materials

The following supporting information can be downloaded at: https://www.mdpi.com/article/10.3390/jcm13226629/s1, Figure S1: Risk of bias graph for clinical trials. Figure S2: Risk of bias summary for clinical trials. Figure S3: Forest plot of the analysis; Cesarean section of suspected fetal distress. Figure S4: Forest plot of the analysis; Cesarean section of non-progressive labor. Figure S5: Forest plot of the analysis; Augmentation of labor. Figure S6: Forest plot of the analysis; NO2 or general anesthesia. Figure S7: Forest plot of the analysis; Local analgesia (pudendal nerve block). Figure S8: Forest plot of the analysis; Narcotics use. Figure S9: Forest plot of the analysis; Acupuncture pain relief. Figure S10: Forest plot of the analysis; Hydrotherapy pain relief. Figure S11: Forest plot of the analysis; No pain relief. Figure S12: Forest plot of the analysis; Episiotomy. Figure S13: Forest plot of the analysis; Physiological management of the third stage of labor. Figure S14: Forest plot of the analysis; Intact perineum. Figure S15: Forest plot of the analysis; First or second-degree perineal tear. Figure S16: Forest plot of the analysis; Third or fourth-degree perineal tear. Figure S17: Forest plot of the analysis; Vaginal or labial tear. Figure S18: Forest plot of the analysis; Manual removal of the placenta Figure S19: Forest plot of the analysis; Blood transfusion. Figure S20: Forest plot of the analysis; Maternal infection or fever. Figure S21: Forest plot of the analysis; Severe maternal morbidity. Figure S22: Forest plot of the analysis; Maternal intensive care unit admission. Figure S23: Forest plot of the analysis; Duration of labor (in hours). Figure S24: Forest plot of the analysis; Duration of hospital stay (in days). Figure S25: Forest plot of the analysis; Mean APGAR score (on-minute and five-minute). Figure S26: Forest plot of the analysis; Umbilical cord arterial pH < 7.1. Figure S27: Forest plot of the analysis; Mean umbilical cord arterial pH. Figure S28: Forest plot of the analysis; Meconium-stained amniotic fluid. Figure S29: Forest plot of the analysis; Asphyxia. Figure S30: Forest plot of the analysis; Need for resuscitation. Figure S31: Forest plot of the analysis; Need for ventilation. Figure S32: Forest plot of the analysis; Shoulder dystocia. Figure S33: Forest plot of the analysis; Transfer to specialist neonatal care. Figure S34: Forest plot of the analysis; Neonatal intensive care unit admission. Figure S35: Forest plot of the analysis; Breastfeeding initiation. Figure S36: Funnel plot of publication bias; Breastfeeding initiation. Figure S37: Funnel plot of publication bias; Episiotomy. Figure S38: Funnel plot of publication bias; Instrumental vaginal delivery. Figure S39: Funnel plot of publication bias; NICU admission. Figure S40: Funnel plot of publication bias; 1st or 2nd degree perineal tear. Figure S41: Funnel plot of publication bias; 3rd or 4th degree perineal tear. Figure S42: Funnel plot of publication bias; Apgar score less than 7. Figure S43: Funnel plot of publication bias; Augmentation of labor. Figure S44: Funnel plot of publication bias; Epidural or Spinal analgesia. Figure S45: Funnel plot of publication bias; Intact perineum. Figure S46: Funnel plot of publication bias; Intrapartum or neonatal mortality. Figure S47: Funnel plot of publication bias; N2O or General analgesia. Figure S48: Funnel plot of publication bias; PPH. Figure S49: Funnel plot of publication bias; Unplanned cesarian delivery. Table S1: Quality assessment of cohort and cross-sectional studies. Table S2: Quality assessment of case-control studies.
