# Supplementary material for: Midwife-Led Versus Obstetrician-Led Perinatal Care for Low-Risk Pregnancy: A Systematic Review and Meta-Analysis of 1.4 Million Pregnancies
Source: J Clin Med. 2024 Nov 5;13(22):6629. doi: 10.3390/jcm13226629 (PMC11594941; doi:10.3390/jcm13226629)
Supplement: Supplementary file 1 [file jcm-13-06629-s001.zip › Supplementary tables.docx]

**Table. S.1:** Quality assessment of cohort and cross-sectional studies.

| **Study ID** | **1. Was the research question or objective in this paper clearly stated?** | **2. Was the study population clearly specified and defined?** | **3. Was the participation rate of eligible persons at least 50%?** | **4. Were all the subjects selected or recruited from the same or similar populations (including the same time period)? Were inclusion and exclusion criteria for being in the study prespecified and applied uniformly to all participants?** | **5. Was a sample size justification, power description, or variance and effect estimates provided?** | **6. For the analyses in this paper, were the exposure(s) of interest measured prior to the outcome(s) being measured?** | **7. Was the time frame sufficient so that one could reasonably expect to see an association between exposure and outcome if it existed?** | **8. For exposures that can vary in amount or level, did the study examine different levels of the exposure as related to the outcome (e.g., categories of exposure, or exposure measured as continuous variable)?** | **9. Were the exposure measures (independent variables) clearly defined, valid, reliable, and implemented consistently across all study participants?** | **10. Was the exposure(s) assessed more than once over time?** | **11. Were the outcome measures prespecified, clearly defined, valid, reliable, and assessed consistently across all study participants?** | **12. Were the people assessing the outcomes blinded to the participants' exposures/interventions?** | **13. Was the loss to follow-up after baseline 20% or less? Were those lost to follow-up accounted for in the analysis?** | **14. Were key potential confounding variables measured and adjusted statistically for their impact on the relationship between exposure(s) and outcome(s)?** | **Total score** | **Quality rating** |
| --- | --- | --- | --- | --- | --- | --- | --- | --- | --- | --- | --- | --- | --- | --- | --- | --- |
| **Sorbara et al. 2024 (1)** | Yes | Yes | Yes | Yes | No | Yes | Yes | NA | Yes | Yes | Yes | NA | Yes | Yes | **11.5** | **Good** |
| **Palau-Costafreda et al. 2023 (2)** | Yes | Yes | Yes | Yes | No | Yes | Yes | NA | Yes | Yes | Yes | NA | Yes | Yes | **11.5** | **Good** |
| **Stoll et al. 2023 (3)** | Yes | Yes | Yes | Yes | No | Yes | Yes | No | Yes | Yes | Yes | NA | Yes | Yes | **12** | **Good** |
| **Martin-Arribas et al. 2022 (4)** | Yes | Yes | Yes | Yes | Yes | Yes | Yes | NA | Yes | Yes | Yes | NA | Yes | Yes | **12** | **Good** |
| **Tietjen et al. 2021 (5)** | Yes | Yes | Yes | Yes | Yes | Yes | Yes | NA | Yes | Yes | Yes | NA | Yes | Yes | **12** | **Good** |
| **Merz et al. 2020 (6)** | Yes | Yes | Yes | Yes | No | Yes | Yes | NA | Yes | Yes | Yes | NA | Yes | Yes | **11.5** | **Good** |
| **Welffens et al. 2019 (7)** | Yes | Yes | Yes | Yes | No | Yes | Yes | NA | Yes | Yes | Yes | NA | Yes | Yes | **11.5** | **Good** |
| **Wiegerinck et al. 2020 (8)** | Yes | Yes | Yes | Yes | No | Yes | Yes | No | Yes | Yes | Yes | NA | Yes | Yes | **12** | **Good** |
| **Isaline et al. 2019 (9)** | Yes | Yes | Yes | Yes | No | Yes | Yes | NA | Yes | Yes | Yes | NA | Yes | Yes | **11.5** | **Good** |
| **Koto et al. 2019 (10)** | Yes | Yes | Yes | Yes | No | Yes | Yes | NA | Yes | Yes | Yes | NA | Yes | Yes | **11.5** | **Good** |
| **Souter et al. 2019 (11)** | Yes | Yes | Yes | Yes | No | Yes | Yes | NA | Yes | Yes | Yes | NA | Yes | Yes | **11.5** | **Good** |
| **Bartuseviciene et al. 2018 (12)** | Yes | Yes | Yes | Yes | No | Yes | Yes | NA | Yes | Yes | Yes | NA | Yes | Yes | **12** | **Good** |
| **Carlson et al. 2018 (13)** | Yes | Yes | Yes | Yes | Yes | Yes | Yes | NA | Yes | Yes | Yes | NA | Yes | Yes | **12** | **Good** |
| **Hua et al. 2018 (14)** | Yes | Yes | Yes | Yes | No | Yes | Yes | Yes | Yes | Yes | Yes | NA | Yes | Yes | **12.5** | **Good** |
| **Wiegerinck et al. 2018 (15)** | Yes | Yes | Yes | Yes | Yes | Yes | Yes | No | Yes | Yes | Yes | NA | Yes | Yes | **12.5** | **Good** |
| **Altman et al. 2017 (16)** | Yes | Yes | Yes | Yes | No | Yes | Yes | NA | Yes | Yes | Yes | NA | Yes | Yes | **11.5** | **Good** |
| **Bodner-Adler et al. 2017 & 2004 (17, 18)** | Yes | Yes | Yes | Yes | No | Yes | Yes | NA | Yes | Yes | Yes | NA | Yes | Yes | **12** | **Good** |
| **Schroeder et al. 2017 (19)** | Yes | Yes | Yes | Yes | No | Yes | Yes | NA | Yes | Yes | Yes | NA | Yes | No | **11** | **Good** |
| **Voon et al. 2017 (20)** | Yes | Yes | Yes | Yes | Yes | Yes | Yes | NA | Yes | Yes | Yes | NA | Yes | Yes | **12** | **Good** |
| **Thiessen et al. 2016 (21)** | Yes | Yes | Yes | Yes | No | Yes | Yes | No | Yes | No | Yes | NA | Yes | Yes | **11.5** | **Good** |
| **Thornton et al. 2016 (22)** | Yes | Yes | Yes | Yes | Yes | Yes | Yes | NA | Yes | Yes | Yes | NA | Yes | Yes | **12** | **Good** |
| **De Jonge et al. 2015 (23)** | Yes | Yes | Yes | Yes | No | Yes | Yes | No | Yes | Yes | Yes | NA | Yes | Yes | **12** | **Good** |
| **Walters et al. 2015 (24)** | Yes | Yes | Yes | Yes | Yes | Yes | Yes | NA | Yes | Yes | Yes | NA | Yes | No | **11.5** | **Good** |
| **Homer et al. 2014 (25)** | Yes | Yes | Yes | Yes | Yes | Yes | Yes | Yes | Yes | Yes | Yes | NA | Yes | Yes | **13** | **Good** |
| **Iida et al. 2014 (26)** | Yes | Yes | Yes | Yes | Yes | Yes | Yes | NA | Yes | Yes | Yes | NA | Yes | No | **11.5** | **Good** |
| **Tracy et al. 2014 (27)** | Yes | Yes | Yes | Yes | No | Yes | Yes | Yes | Yes | No | Yes | NA | Yes | No | **11.5** | **Good** |
| **Hiraizumi et al. 2013 (28)** | Yes | Yes | Yes | Yes | No | Yes | Yes | No | Yes | Yes | Yes | NA | Yes | No | **11.5** | **Good** |
| **Burns et al. 2012 (29)** | Yes | Yes | Yes | Yes | Yes | Yes | Yes | Yes | Yes | Yes | Yes | NA | Yes | No | **12.5** | **Good** |
| **Brocklehurst et al. 2011 (30)** | Yes | Yes | Yes | Yes | Yes | Yes | Yes | Yes | Yes | Yes | Yes | NA | Yes | Yes | **13** | **Good** |
| **Davis et al. 2011 & 2012 (31, 32)** | Yes | Yes | Yes | Yes | No | Yes | Yes | Yes | Yes | Yes | Yes | NA | Yes | Yes | **12.5** | **Good** |
| **Gottvall et al. 2011 (33)** | Yes | Yes | Yes | Yes | Yes | Yes | Yes | NA | Yes | Yes | Yes | NA | Yes | Yes | **12** | **Good** |
| **Overgaard et al. 2011 (34)** | Yes | Yes | Yes | Yes | Yes | Yes | Yes | NA | Yes | Yes | Yes | NA | Yes | Yes | **12** | **Good** |
| **Browne et al. 2010 (35)** | Yes | Yes | Yes | Yes | No | Yes | Yes | NA | Yes | No | Yes | NA | Yes | Yes | **11** | **Good** |
| **Eide et al. 2009 (36)** | Yes | Yes | Yes | Yes | Yes | Yes | Yes | NA | Yes | Yes | Yes | NA | Yes | Yes | **12** | **Good** |
| **Suzuki et al. 2009 (37)** | Yes | Yes | Yes | Yes | No | Yes | Yes | NA | Yes | Yes | Yes | NA | Yes | No | **11** | **Good** |
| **Maassen et al. 2008 (38)** | Yes | Yes | Yes | Yes | No | Yes | Yes | No | Yes | Yes | Yes | NA | Yes | No | **11.5** | **Good** |
| **Ryan et al. 2005 (39)** | Yes | Yes | Yes | Yes | No | Yes | Yes | NA | Yes | Yes | Yes | NA | Yes | No | **12** | **Good** |
| **Rana et al. 2003 (40)** | Yes | No | Yes | Yes | Yes | Yes | Yes | NA | Yes | Yes | Yes | NA | Yes | No | **11** | **Good** |

*Each question is answered: Yes=1, No=0.5, Not Reported (NR), Cannot Determine (CD) or Not Applicable (NA)=0*

**Table. S.2:** Quality assessment of case-control studies.

| **Study ID** | **1. Was the research question or objective in this paper clearly stated and appropriate?** | **2. Was the study population clearly specified and defined?** | **3. Did the authors include a sample size justification?** | **4. Were controls selected or recruited from the same or similar population that gave rise to the cases (including the same timeframe)?** | **5. Were the definitions, inclusion and exclusion criteria, algorithms or processes used to identify or select cases and controls valid, reliable, and implemented consistently across all study participants?** | **6. Were the cases clearly defined and differentiated from controls?** | **7. If less than 100 percent of eligible cases and/or controls were selected for the study, were the cases and/or controls randomly selected from those eligible?** | **8. Was there use of concurrent controls?** | **9. Were the investigators able to confirm that the exposure/risk occurred prior to the development of the condition or event that defined a participant as a case?** | **10. Were the measures of exposure/risk clearly defined, valid, reliable, and implemented consistently (including the same time period) across all study participants?** | **11. Were the assessors of exposure/risk blinded to the case or control status of participants?** | **12. Were key potential confounding variables measured and adjusted statistically in the analyses? If matching was used, did the investigators account for matching during study analysis?** | **Total score** | **Quality rating** |
| --- | --- | --- | --- | --- | --- | --- | --- | --- | --- | --- | --- | --- | --- | --- |
| **Poskienc et al. 2021 (41)** | Yes | Yes | No | Yes | Yes | Yes | NA | No | NA | Yes | NA | Yes | 8 | Fair |
| **Prelec et al. 2014 (42)** | Yes | Yes | Yes | Yes | Yes | Yes | NA | No | NA | Yes | NA | No | 8 | Fair |
| **Gaudineau et al. 2012 (43)** | Yes | Yes | No | Yes | Yes | Yes | Yes | No | NA | Yes | NA | Yes | 9 | Fair |

*Each question is answered: Yes=1, No=0.5, Not Reported (NR), Cannot Determine (CD) or Not Applicable (NA)=0*

**References**

1. Sorbara C, Ray JG, Darling EK, Chung H, Podolsky S, Stukel TA. Postpartum Emergency Department Use Following Midwifery-Model vs Obstetrics-Model Care. JAMA network open. 2024;7(4):e248676.

2. Palau-Costafreda R, García Gumiel S, Eles Velasco A, et al. The first alongside midwifery unit in Spain: A retrospective cohort study of maternal and neonatal outcomes. Birth (Berkeley, Calif). 2023;50(4):1057-67.

3. Stoll K, Titoria R, Turner M, Jones A, Butska L. Perinatal outcomes of midwife-led care, stratified by medical risk: a retrospective cohort study from British Columbia (2008-2018). CMAJ : Canadian Medical Association journal = journal de l'Association medicale canadienne. 2023;195(8):E292-e9.

4. Martin-Arribas A, Escuriet R, Borràs-Santos A, Vila-Candel R, González-Blázquez C. A comparison between midwifery and obstetric care at birth in Spain: Across-sectional study of perinatal outcomes. International journal of nursing studies. 2022;126:104129.

5. Tietjen SL, Schmitz MT, Heep A, et al. Model of care and chance of spontaneous vaginal birth: a prospective, multicenter matched-pair analysis from North Rhine-Westphalia. BMC Pregnancy Childbirth. 2021;21(1):849.

6. Merz WM, Tascon-Padron L, Puth MT, et al. Maternal and neonatal outcome of births planned in alongside midwifery units: a cohort study from a tertiary center in Germany. BMC Pregnancy Childbirth. 2020;20(1):267.

7. Welffens K, Derisbourg S, Costa E, et al. The "Cocoon," first alongside midwifery-led unit within a Belgian hospital: Comparison of the maternal and neonatal outcomes with the standard obstetric unit over 2 years. Birth (Berkeley, Calif). 2020;47(1):115-22.

8. Wiegerinck MMJ, Eskes M, van der Post JAM, Mol BW, Ravelli ACJ. Intrapartum and neonatal mortality in low-risk term women in midwife-led care and obstetrician-led care at the onset of labor: A national matched cohort study. Acta obstetricia et gynecologica Scandinavica. 2020;99(4):546-54.

9. Isaline G, Marie-Christine C, Rudy VT, Caroline D, Yvon E. An exploratory cost-effectiveness analysis: Comparison between a midwife-led birth unit and a standard obstetric unit within the same hospital in Belgium. Midwifery. 2019;75:117-26.

10. Koto PS, Fahey J, Meier D, LeDrew M, Loring S. Relative effectiveness and cost-effectiveness of the midwifery-led care in Nova Scotia, Canada: A retrospective, cohort study. Midwifery. 2019;77:144-54.

11. Souter V, Nethery E, Kopas ML, Wurz H, Sitcov K, Caughey AB. Comparison of Midwifery and Obstetric Care in Low-Risk Hospital Births. Obstetrics and gynecology. 2019;134(5):1056-65.

12. Bartuseviciene E, Kacerauskiene J, Bartusevicius A, et al. Comparison of midwife-led and obstetrician-led care in Lithuania: A retrospective cohort study. Midwifery. 2018;65:67-71.

13. Carlson NS, Corwin EJ, Hernandez TL, Holt E, Lowe NK, Hurt KJ. Association between provider type and cesarean birth in healthy nulliparous laboring women: A retrospective cohort study. Birth (Berkeley, Calif). 2018;45(2):159-68.

14. Hua J, Zhu L, Du L, et al. Effects of midwife-led maternity services on postpartum wellbeing and clinical outcomes in primiparous women under China's one-child policy. BMC Pregnancy Childbirth. 2018;18(1):329.

15. Wiegerinck MMJ, van der Goes BY, Ravelli ACJ, et al. Intrapartum and neonatal mortality among low-risk women in midwife-led versus obstetrician-led care in the Amsterdam region of the Netherlands: a propensity score matched study. BMJ open. 2018;8(1):e018845.

16. Altman MR, Murphy SM, Fitzgerald CE, Andersen HF, Daratha KB. The Cost of Nurse-Midwifery Care: Use of Interventions, Resources, and Associated Costs in the Hospital Setting. Women's health issues : official publication of the Jacobs Institute of Women's Health. 2017;27(4):434-40.

17. Bodner-Adler B, Kimberger O, Griebaum J, Husslein P, Bodner K. A ten-year study of midwife-led care at an Austrian tertiary care center: a retrospective analysis with special consideration of perineal trauma. BMC Pregnancy Childbirth. 2017;17(1):357.

18. Bodner-Adler B, Bodner K, Kimberger O, Lozanov P, Husslein P, Mayerhofer K. Influence of the birth attendant on maternal and neonatal outcomes during normal vaginal delivery: a comparison between midwife and physician management. Wiener klinische Wochenschrift. 2004;116(11-12):37984.

19. Schroeder L, Patel N, Keeler M, Rocca-Ihenacho L, Macfarlane AJ. The economic costs of intrapartum care in Tower Hamlets: A comparison between the cost of birth in a freestanding midwifery unit and hospital for women at low risk of obstetric complications. Midwifery. 2017;45:28-35.

20. Voon ST, Lay JTS, San WTW, Shorey S, Lin SKS. Comparison of midwife-led care and obstetrician-led care on maternal and neonatal outcomes in Singapore: A retrospective cohort study. Midwifery. 2017;53:71-9.

21. Thiessen K, Nickel N, Prior HJ, Banerjee A, Morris M, Robinson K. Maternity Outcomes in Manitoba Women: A Comparison between Midwifery-led Care and Physician-led Care at Birth. Birth (Berkeley, Calif). 2016;43(2):108-15.

22. Thornton P, McFarlin BL, Park C, et al. Cesarean Outcomes in US Birth Centers and Collaborating Hospitals: A Cohort Comparison. Journal of midwifery & women's health. 2017;62(1):40-8.

23. de Jonge A, Mesman JA, Manniën J, et al. Severe Adverse Maternal Outcomes among Women in Midwife-Led versus Obstetrician-Led Care at the Onset of Labour in the Netherlands: A Nationwide Cohort Study. PloS one. 2015;10(5):e0126266.

24. Walters D, Gupta A, Nam AE, Lake J, Martino F, Coyte PC. A Cost-Effectiveness Analysis of Low-Risk Deliveries: A Comparison of Midwives, Family Physicians and Obstetricians. Healthcare policy = Politiques de sante. 2015;11(1):61-75.

25. Homer CS, Thornton C, Scarf VL, et al. Birthplace in New South Wales, Australia: an analysis of perinatal outcomes using routinely collected data. BMC Pregnancy Childbirth. 2014;14:206.

26. Iida M, Horiuchi S, Nagamori K. A comparison of midwife-led care versus obstetrician-led care for low-risk women in Japan. Women and birth : journal of the Australian College of Midwives. 2014;27(3):202-7.

27. Tracy SK, Welsh A, Hall B, et al. Caseload midwifery compared to standard or private obstetric care for first time mothers in a public teaching hospital in Australia: a cross sectional study of cost and birth outcomes. BMC Pregnancy Childbirth. 2014;14:46.

28. Hiraizumi Y, Suzuki S. Perinatal outcomes of low-risk planned home and hospital births under midwife-led care in Japan. The journal of obstetrics and gynaecology research. 2013;39(11):1500-4.

29. Burns EE, Boulton MG, Cluett E, Cornelius VR, Smith LA. Characteristics, interventions, and outcomes of women who used a birthing pool: a prospective observational study. Birth (Berkeley, Calif). 2012;39(3):192-202.

30. Brocklehurst P, Hardy P, Hollowell J, et al. Perinatal and maternal outcomes by planned place of birth for healthy women with low risk pregnancies: the Birthplace in England national prospective cohort study. BMJ (Clinical research ed). 2011;343:d7400.

31. Davis D, Baddock S, Pairman S, et al. Risk of severe postpartum hemorrhage in low-risk childbearing women in new zealand: exploring the effect of place of birth and comparing third stage management of labor. Birth (Berkeley, Calif). 2012;39(2):98-105.

32. Davis D, Baddock S, Pairman S, et al. Planned place of birth in New Zealand: does it affect mode of birth and intervention rates among low-risk women? Birth (Berkeley, Calif). 2011;38(2):111-9.

33. Gottvall K, Waldenström U, Tingstig C, Grunewald C. In-hospital birth center with the same medical guidelines as standard care: a comparative study of obstetric interventions and outcomes. Birth (Berkeley, Calif). 2011;38(2):120-8.

34. Overgaard C, Møller AM, Fenger-Grøn M, Knudsen LB, Sandall J. Freestanding midwifery unit versus obstetric unit: a matched cohort study of outcomes in low-risk women. BMJ open. 2011;1(2):e000262.

35. Browne M, Jacobs M, Lahiff M, Miller S. Perineal injury in nulliparous women giving birth at a community hospital: reduced risk in births attended by certified nurse-midwives. Journal of midwifery & women's health. 2010;55(3):243-9.

36. Eide BI, Nilsen AB, Rasmussen S. Births in two different delivery units in the same clinic--a prospective study of healthy primiparous women. BMC Pregnancy Childbirth. 2009;9:25.

37. Suzuki S, Satomi M, Miyake H. Referrals during labor in midwifery care. Journal of Nippon Medical School = Nippon Ika Daigaku zasshi. 2009;76(4):226-8.

38. Maassen MS, Hendrix MJ, Van Vugt HC, Veersema S, Smits F, Nijhuis JG. Operative deliveries in low-risk pregnancies in The Netherlands: primary versus secondary care. Birth (Berkeley, Calif). 2008;35(4):277-82.

39. Ryan M, Roberts C. A retrospective cohort study comparing the clinical outcomes of a birth centre and labour ward in the same hospital. Australian Midwifery. 2005;18(2):17-21.

40. Rana TG, Rajopadhyaya R, Bajracharya B, Karmacharya M, Osrin D. Comparison of midwifery-led and consultant-led maternity care for low risk deliveries in Nepal. Health policy and planning. 2003;18(3):330-7.

41. Poškienė I, Vanagas G, Kirkilytė A, Nadišauskienė RJ. Comparison of vaginal birth outcomes in midwifery-led versus physician-led setting: A propensity score-matched analysis. Open medicine (Warsaw, Poland). 2021;16(1):1537-43.

42. Prelec A, Verdenik I, Poat A. A comparison of frequency of medical interventions and birth outcomes between the midwife led unit and the obstetric unit in low-risk primiparous women. Slovenian Nursing Review. 2014;48.

43. Gaudineau A, Sauleau EA, Nisand I, Langer B. Obstetric and neonatal outcomes in a home-like birth centre: a case-control study. Archives of gynecology and obstetrics. 2013;287(2):211-6.
