# Supplementary figures and images for: Midwife-Led Versus Obstetrician-Led Perinatal Care for Low-Risk Pregnancy: A Systematic Review and Meta-Analysis of 1.4 Million Pregnancies
Source: J Clin Med. 2024 Nov 5;13(22):6629. doi: 10.3390/jcm13226629 (PMC11594941; doi:10.3390/jcm13226629)

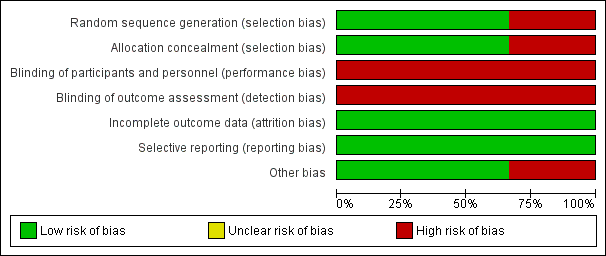

Supplement: Supplementary file 1 [file jcm-13-06629-s001.zip › Figure S.1 Risk of bias graph for RCTS.png]

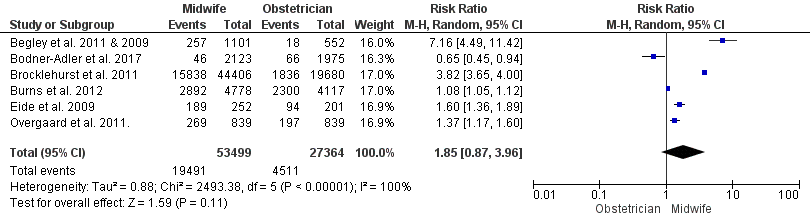

Supplement: Supplementary file 1 [file jcm-13-06629-s001.zip › Figure S.10. Hydrotherapy pain relief.png]

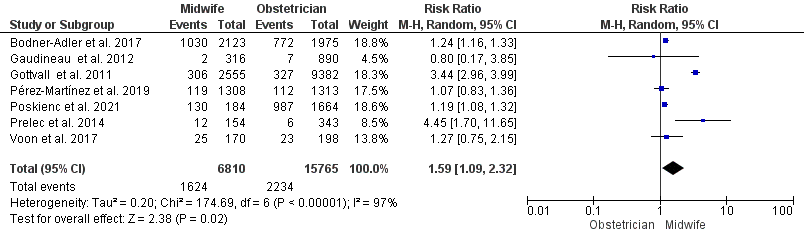

Supplement: Supplementary file 1 [file jcm-13-06629-s001.zip › Figure S.11. No pain relief.png]

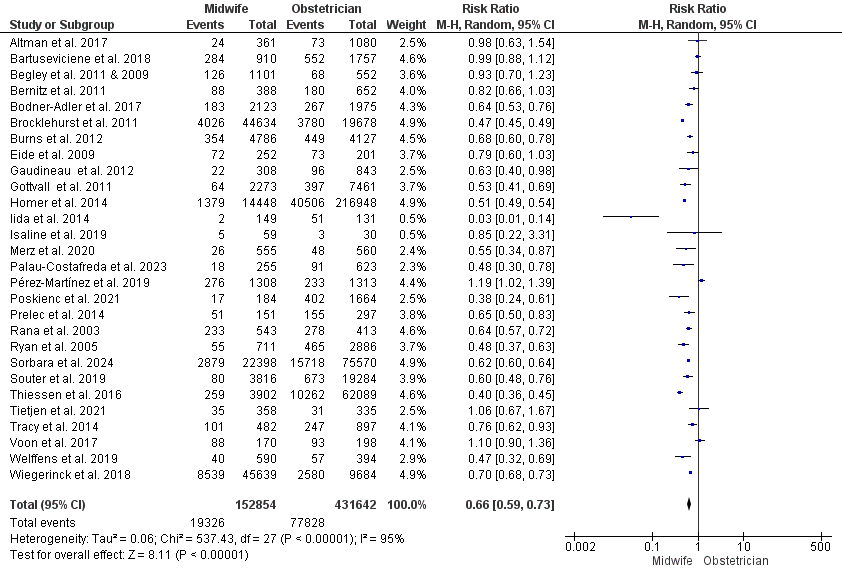

Supplement: Supplementary file 1 [file jcm-13-06629-s001.zip › Figure S.12. Episiotomy.png]

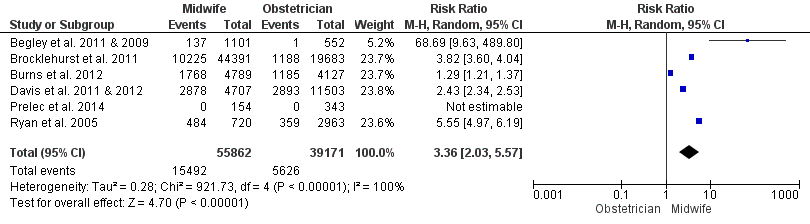

Supplement: Supplementary file 1 [file jcm-13-06629-s001.zip › Figure S.13. Physiological management of 3rd stage of labor.png]

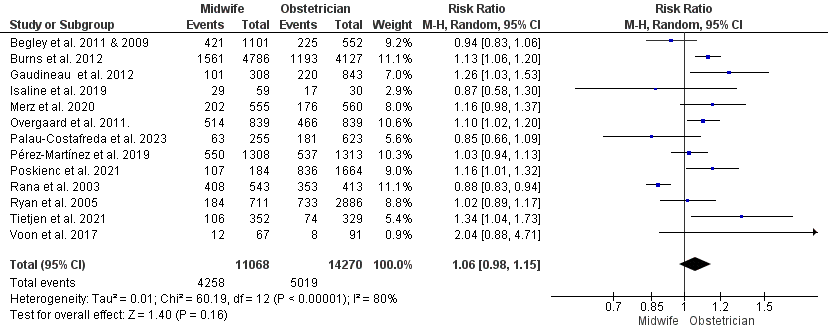

Supplement: Supplementary file 1 [file jcm-13-06629-s001.zip › Figure S.14. Intact perineum.png]

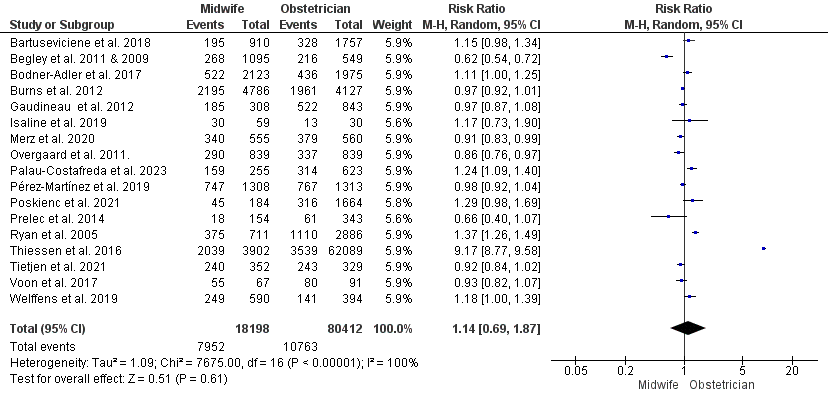

Supplement: Supplementary file 1 [file jcm-13-06629-s001.zip › Figure S.15. 1st or 2nd degree perineal tear.png]

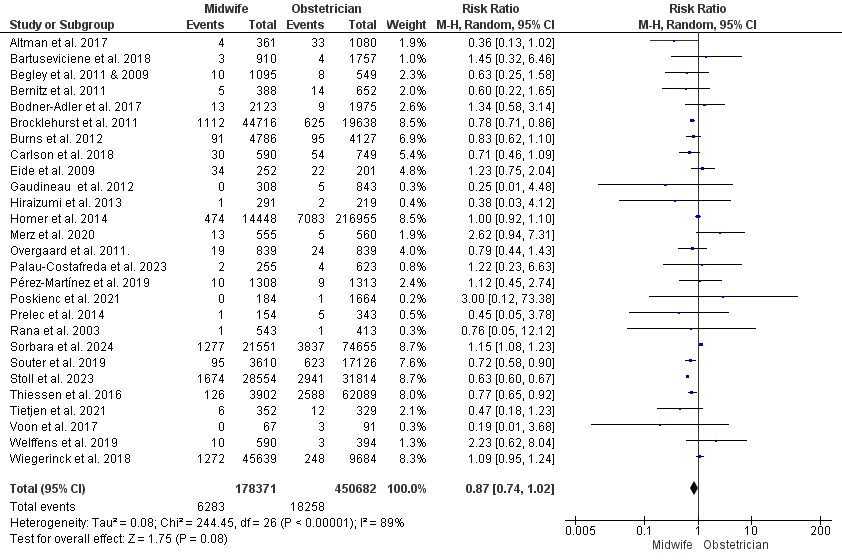

Supplement: Supplementary file 1 [file jcm-13-06629-s001.zip › Figure S.16. 3rd or 4th degree perineal tear.png]

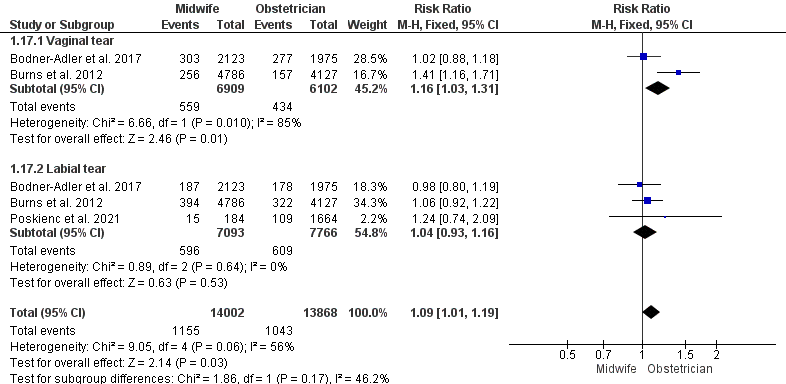

Supplement: Supplementary file 1 [file jcm-13-06629-s001.zip › Figure S.17. Vaginal or Labial tear.png]

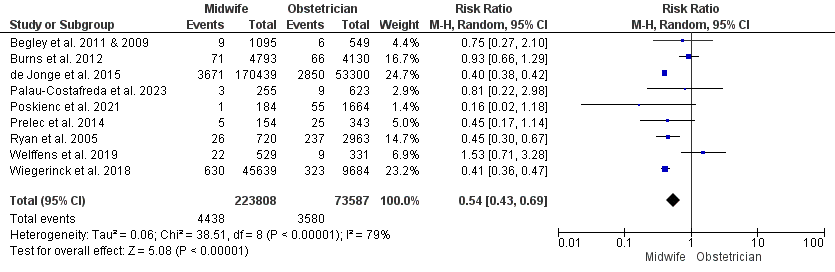

Supplement: Supplementary file 1 [file jcm-13-06629-s001.zip › Figure S.18. Manual removal of placenta.png]

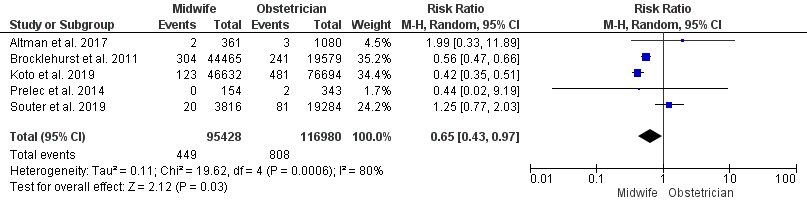

Supplement: Supplementary file 1 [file jcm-13-06629-s001.zip › Figure S.19. Blood transfusion.png]

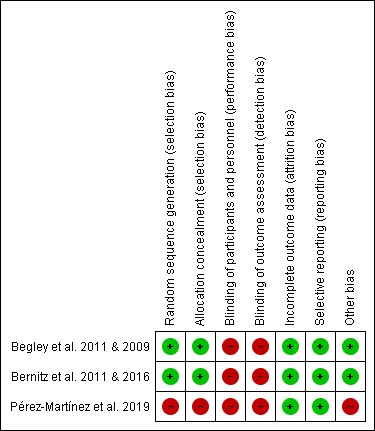

Supplement: Supplementary file 1 [file jcm-13-06629-s001.zip › Figure S.2 Risk of bias summary for RCTs.png]

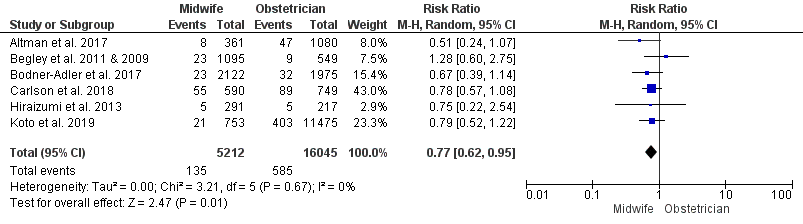

Supplement: Supplementary file 1 [file jcm-13-06629-s001.zip › Figure S.20. Maternal infection orfever.png]

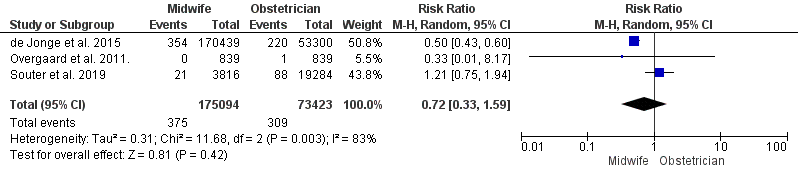

Supplement: Supplementary file 1 [file jcm-13-06629-s001.zip › Figure S.21. Severe maternal morbidity.png]

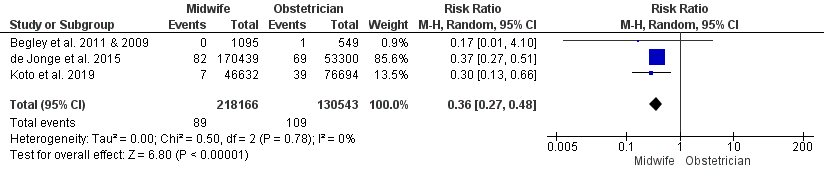

Supplement: Supplementary file 1 [file jcm-13-06629-s001.zip › Figure S.22. Maternal icu admission.png]

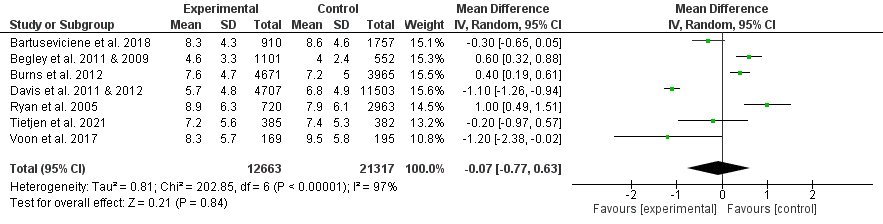

Supplement: Supplementary file 1 [file jcm-13-06629-s001.zip › Figure S.23. Duration of labor (h) .png]

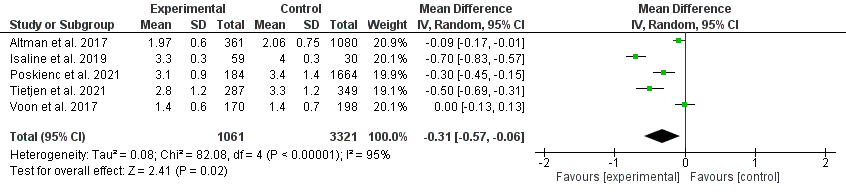

Supplement: Supplementary file 1 [file jcm-13-06629-s001.zip › Figure S.24. Hospital stay duration (days).png]

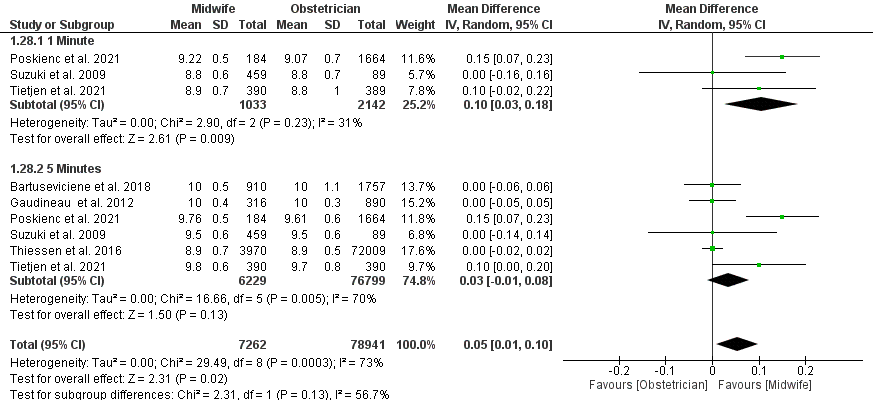

Supplement: Supplementary file 1 [file jcm-13-06629-s001.zip › Figure S.25. Apgar score.png]

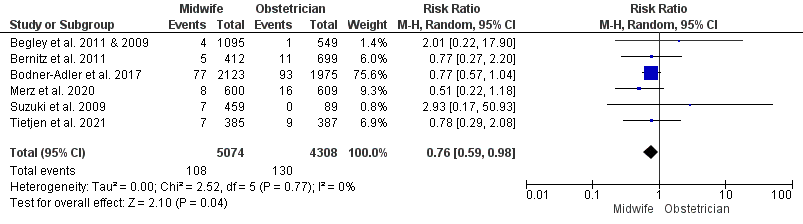

Supplement: Supplementary file 1 [file jcm-13-06629-s001.zip › Figure S.26 Umblical corrd arterial PH less than 7.png]

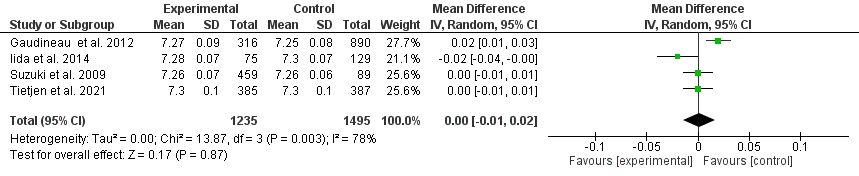

Supplement: Supplementary file 1 [file jcm-13-06629-s001.zip › Figure S.27. Umbilical cord arterial pH.png]

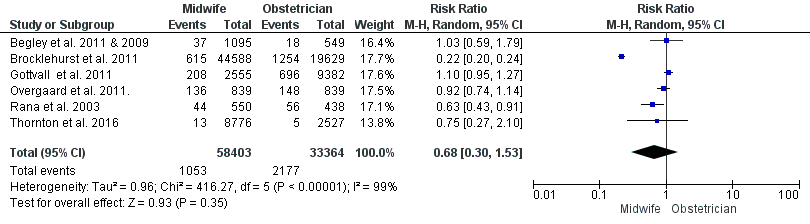

Supplement: Supplementary file 1 [file jcm-13-06629-s001.zip › Figure S.28. Meconium staind fluid.png]

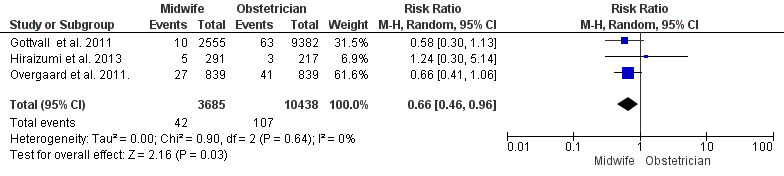

Supplement: Supplementary file 1 [file jcm-13-06629-s001.zip › Figure S.29. Asphyxia.png]

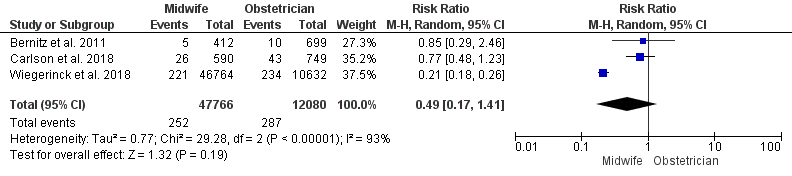

Supplement: Supplementary file 1 [file jcm-13-06629-s001.zip › Figure S.3. CS of Suspected fetal distress.png]

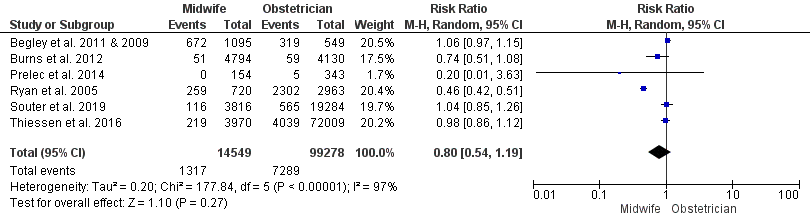

Supplement: Supplementary file 1 [file jcm-13-06629-s001.zip › Figure S.30. Need for resuscitation.png]

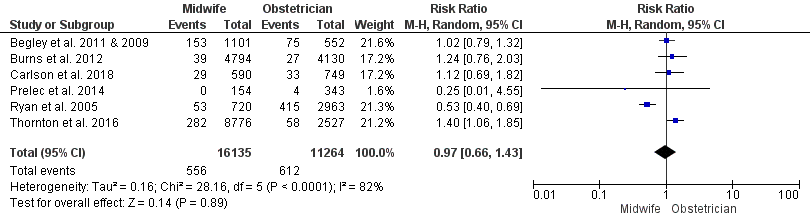

Supplement: Supplementary file 1 [file jcm-13-06629-s001.zip › Figure S.31. Need for ventilation.png]

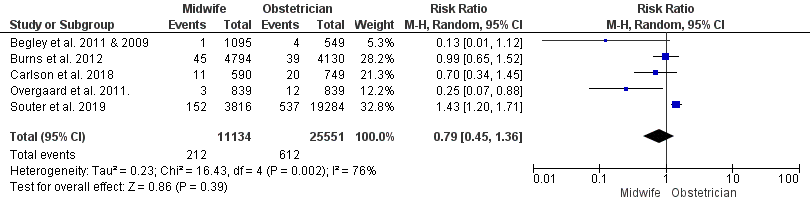

Supplement: Supplementary file 1 [file jcm-13-06629-s001.zip › Figure S.32. Shoulder dystocia.png]

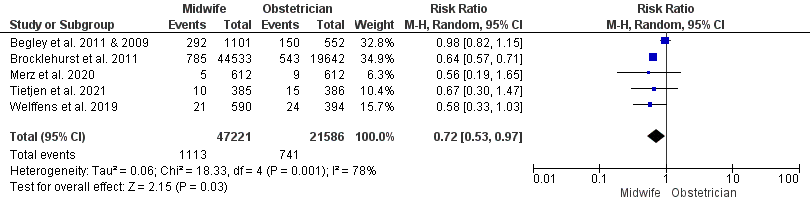

Supplement: Supplementary file 1 [file jcm-13-06629-s001.zip › Figure S.33. Transfer to specialist neonatal care.png]

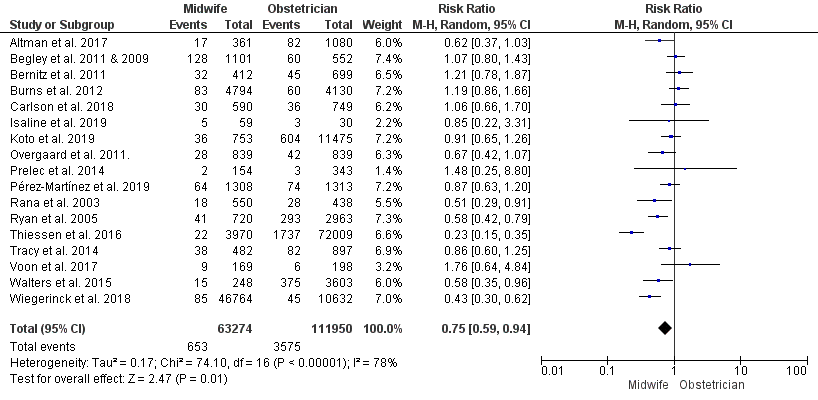

Supplement: Supplementary file 1 [file jcm-13-06629-s001.zip › Figure S.34 NICU admission.png]

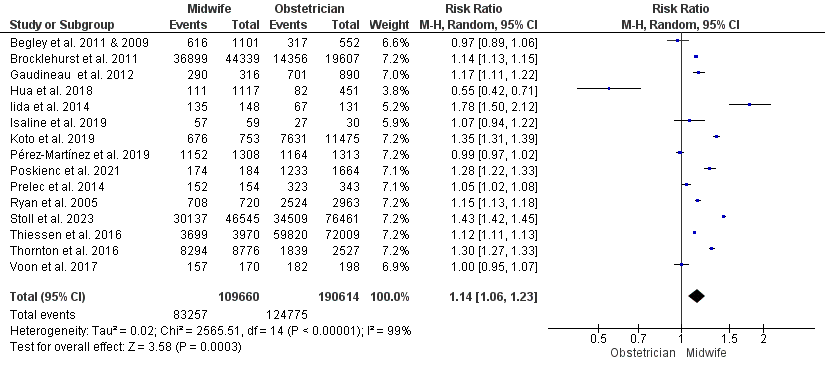

Supplement: Supplementary file 1 [file jcm-13-06629-s001.zip › Figure S.35. Breastfeeding initiation.png]

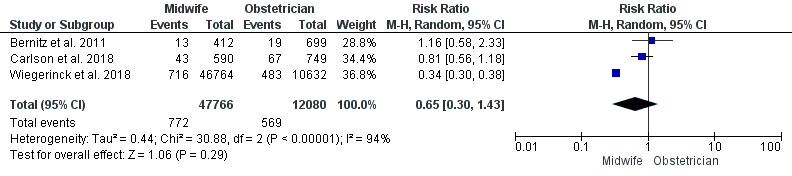

Supplement: Supplementary file 1 [file jcm-13-06629-s001.zip › Figure S.4. CS of Non-progressive labour.png]

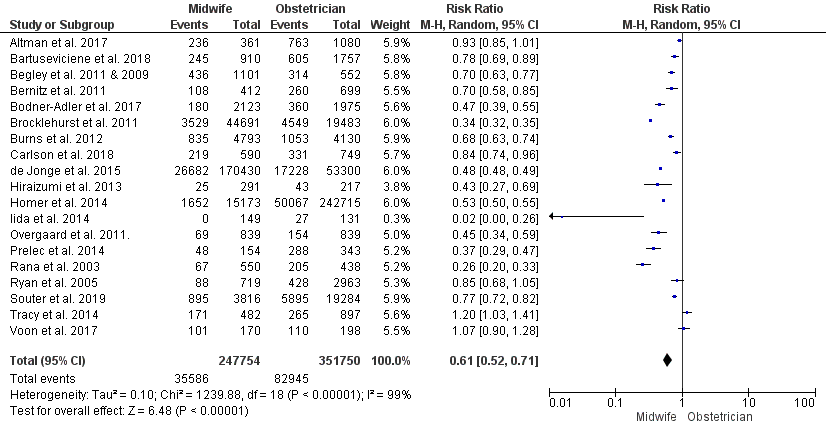

Supplement: Supplementary file 1 [file jcm-13-06629-s001.zip › Figure S.5. Augmentation of labor.png]

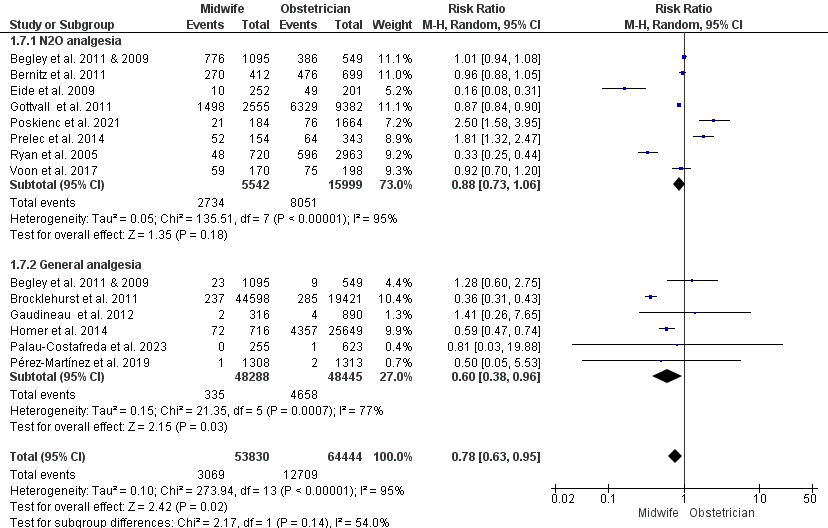

Supplement: Supplementary file 1 [file jcm-13-06629-s001.zip › Figure S.6. N2O or General analgesia.png]

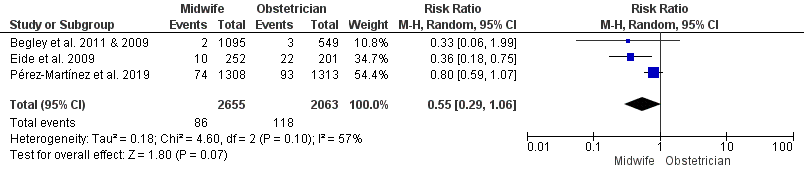

Supplement: Supplementary file 1 [file jcm-13-06629-s001.zip › Figure S.7. local analgesia OR pudendal block.png]

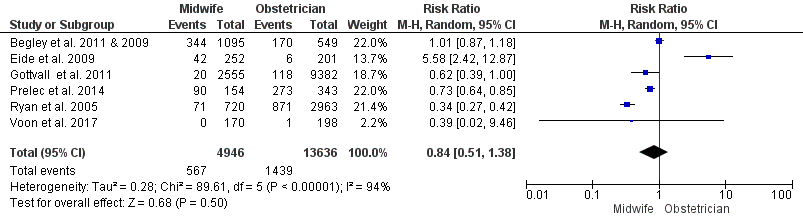

Supplement: Supplementary file 1 [file jcm-13-06629-s001.zip › Figure S.8. Narcotics use.png]

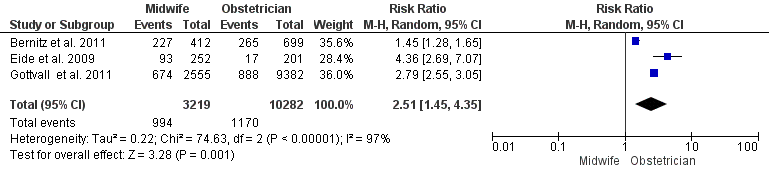

Supplement: Supplementary file 1 [file jcm-13-06629-s001.zip › Figure S.9. Acupuncture pain relief.png]

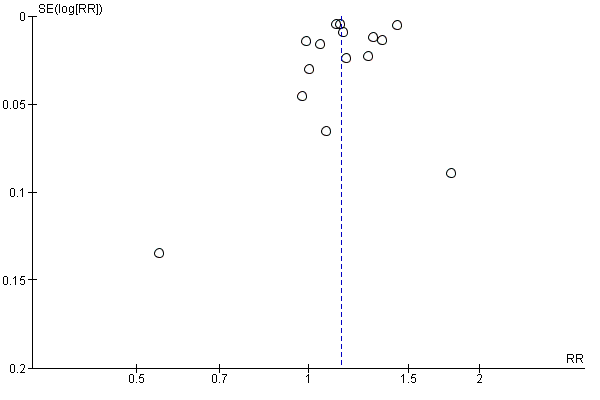

Supplement: Supplementary file 1 [file jcm-13-06629-s001.zip › Figure S36. Publication bias of Breastfeeding initiation.png]

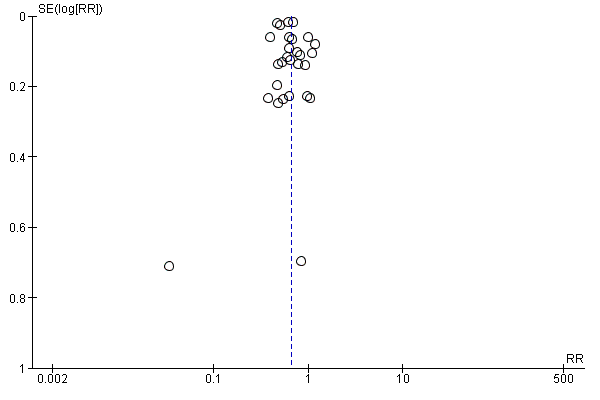

Supplement: Supplementary file 1 [file jcm-13-06629-s001.zip › Figure S37. Publication bias of Episiotomy.png]

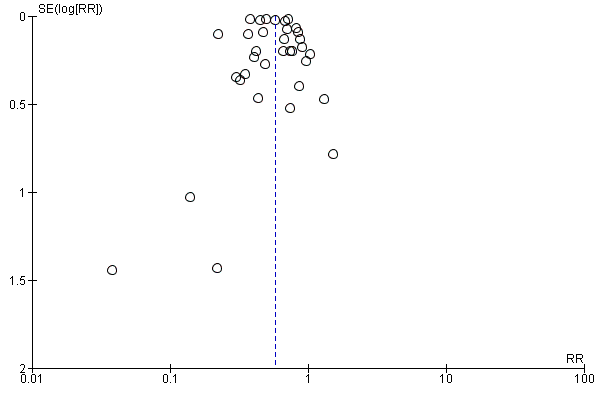

Supplement: Supplementary file 1 [file jcm-13-06629-s001.zip › Figure S38. Publication bias of Instrumental vaginal delivery.png]

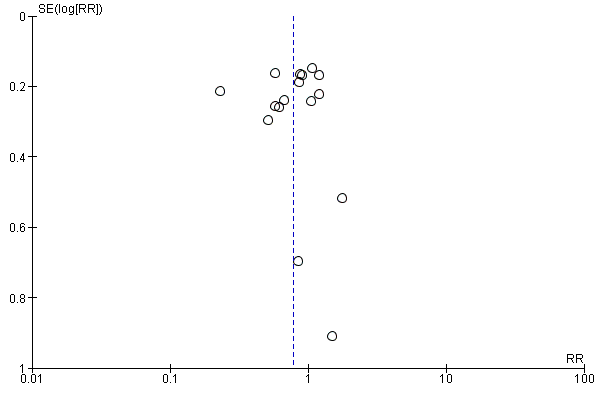

Supplement: Supplementary file 1 [file jcm-13-06629-s001.zip › Figure S39. Publication bias of NICU admission.png]

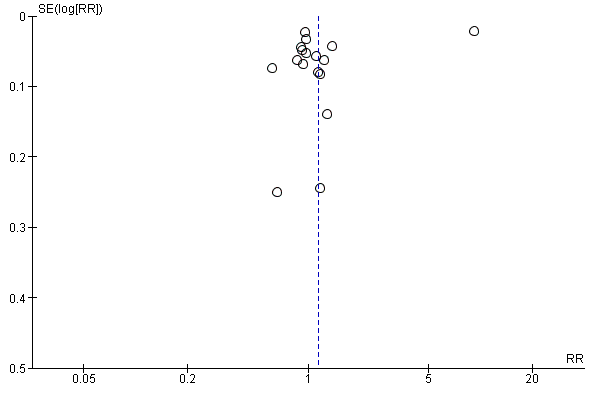

Supplement: Supplementary file 1 [file jcm-13-06629-s001.zip › Figure S40. Publication bias of 1st or 2nd degree perineal tear.png]

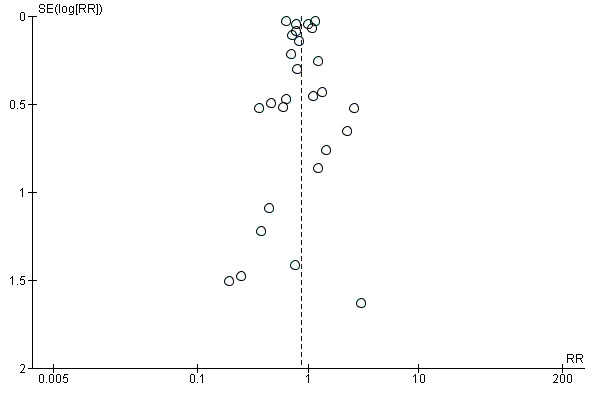

Supplement: Supplementary file 1 [file jcm-13-06629-s001.zip › Figure S41. Publication bias of 3rd or 4th degree perineal tear.png]

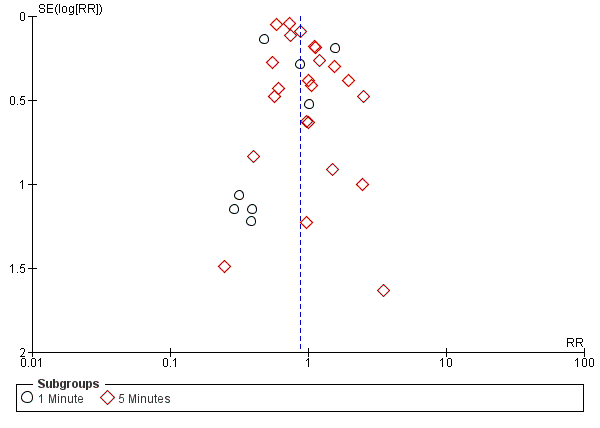

Supplement: Supplementary file 1 [file jcm-13-06629-s001.zip › Figure S42. Publication bias of Apgar score less than 7.png]

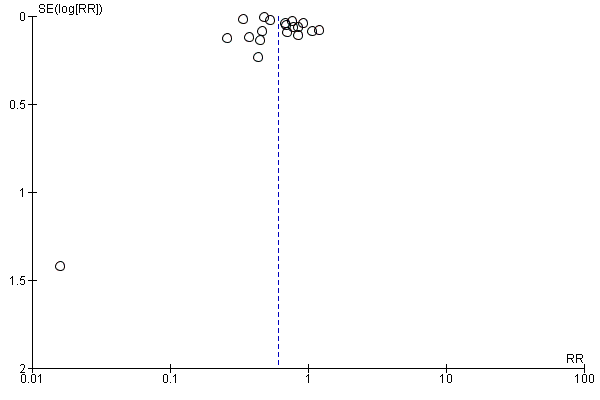

Supplement: Supplementary file 1 [file jcm-13-06629-s001.zip › Figure S43. Publication bias of Augmentation of labor.png]

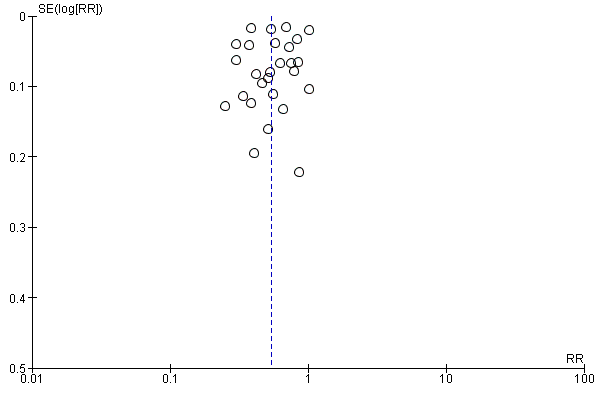

Supplement: Supplementary file 1 [file jcm-13-06629-s001.zip › Figure S44. Publication bias of Epidural or Spinal analgesia.png]

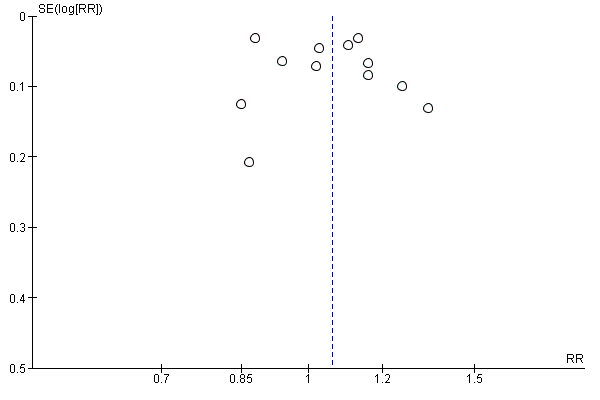

Supplement: Supplementary file 1 [file jcm-13-06629-s001.zip › Figure S45. Publication bias of Intact perineum.png]

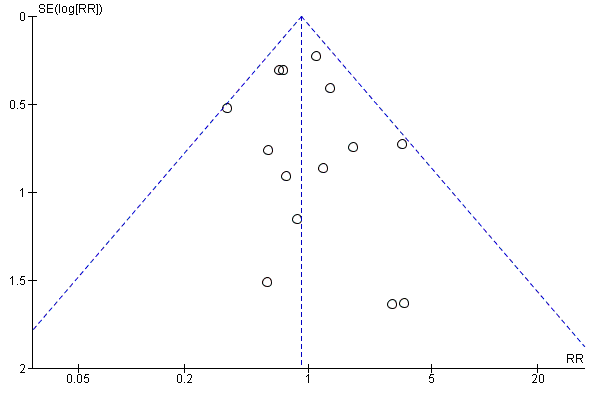

Supplement: Supplementary file 1 [file jcm-13-06629-s001.zip › Figure S46. Publication bias of Intrapartum or neonatal mortality.png]

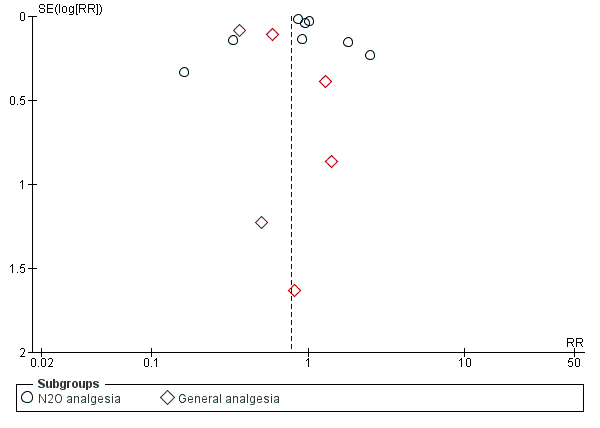

Supplement: Supplementary file 1 [file jcm-13-06629-s001.zip › Figure S47. Publication bias of N2O or General analgesia.png]

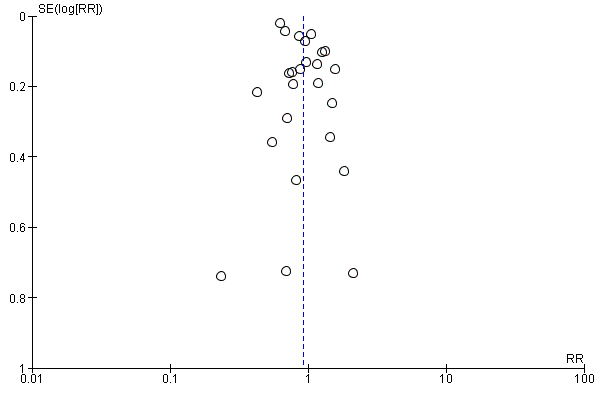

Supplement: Supplementary file 1 [file jcm-13-06629-s001.zip › Figure S48. Publication bias of PPH.png]

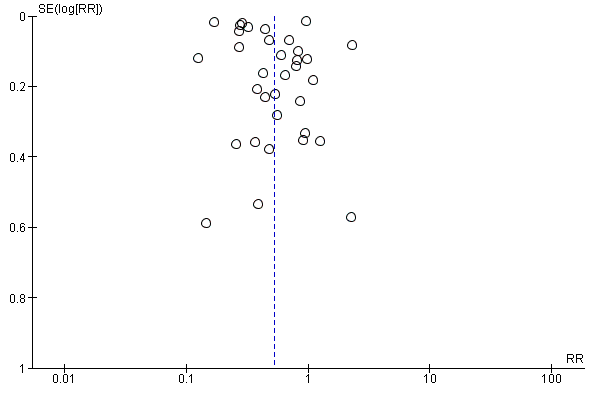

Supplement: Supplementary file 1 [file jcm-13-06629-s001.zip › Figure S49. Publication bias of Unplanned caesarian delivery.png]
